# In vitro localization of modified zinc oxide nanoparticles showing selective anticancer effects against colorectal carcinoma using biophysical techniques

**DOI:** 10.1038/s41598-025-00434-3

**Published:** 2025-05-14

**Authors:** Nouran Al-Shehaby, Hisham A. Elshoky, Mona Zidan, Taher A. Salaheldin, Mohamed H. Gaber, Maha A. Ali, Nayera M. El-Sayed

**Affiliations:** 1 Tumor Biology Research Program, Basic Research Unit, Research Department, Children’s Cancer Hospital Egypt 57357, Cairo, 11441 Egypt; 2 Nanotechnology and Advanced Materials Central Lab, Agricultural Research Center, P.O. 588 Orman, 9 Elgamaa St., Giza, 12619 Egypt; 3 Regional Center for Food and Feed, Agricultural Research Center, Giza, 12619 Egypt; 4 Immunology research program, Basic Research Unit, Research Department, Children’s Cancer Hospital Egypt 57357, Cairo, 11441 Egypt; 5 SUNY Schenectady Community College, New York, USA; 6 Biophysics Department, Faculty of Science, Cairo University, Giza, 12613 Egypt; 7 Physics Department, Faculty of Science, Mansoura University, Mansoura, 35516 Egypt

**Keywords:** Zinc oxide nanoparticles, Tetraethoxysilane, Bovine serum albumin, Caco-2 cell lines, Cytotoxicity, Cellular uptake, Selective anticancer activity., Biotechnology, Cancer

## Abstract

In recent decades, despite advancements in conventional cancer therapies, their serious side effects on both healthy and tumor cells remain a major concern. Aiming to address indiscriminate drug distribution, unwanted toxicity, and high chemotherapy doses, this study explores the targeted delivery of zinc oxide nanoparticles (ZnO NPs). ZnO NPs were synthesized and coated with bovine serum albumin (BSA) and tetraethoxysilane (TEOS) to control cellular uptake and enhance anticancer activity. Characterized by UV-visible spectroscopy, DLS, FTIR, XRD, and TEM, ZnO, ZnOB, and ZnOT particles displayed sizes of 140 ± 13.6 nm, 342 ± 8.4 nm, and 145 ± 23.8 nm, respectively, with ZnOT showing a positive charge of + 19.3 ± 4.16 mV, enhancing stability and cellular interaction. Cytotoxicity assays revealed ZnO’s potent anticancer effect in Caco-2 cells with an IC50 of 219 µg/ml, while ZnOB and ZnOT showed moderate toxicity (IC50 values of 308 µg/ml and 235 µg/ml). HepG2 cells maintained viability close to 100%, highlighting ZnO NPs’ selectivity for Caco-2 cells. Flow cytometry and confocal microscopy indicated differential uptake, with ZnOB showing the highest uptake in Caco-2 cells after 24 h at 37 °C, increasing fluorescence intensity by over 80% compared to ZnO. ZnOT notably increased late apoptotic cells by 65% in Caco-2 lines and caused a 40% rise in G2/M phase arrest. Mitochondrial function assays showed that ZnO reduced mitochondrial membrane potential by over 30%, indicating stress induction. These results support the potential of ZnO-based nanoparticles in colorectal cancer treatment, offering selective cytotoxicity, enhanced cellular uptake, and clear apoptotic activity, making them a promising alternative to conventional chemotherapy.

## Introduction

Cancer remains a major cause of death globally and a significant obstacle to increasing life expectancy. The number of new cancer cases is expected to reach 29.5 million per year^1^. A World Health Organization (WHO) report indicates that cancer deaths are projected to increase from 10 million in 2020 to 16.4 million annually by 2040^2^. Cancer arises from the uncontrolled proliferation of abnormal cells that disregard the signals regulating normal cell behavior, eventually invading other parts of the body^3^. Cancer has usually been treated by chemotherapy, radiation, and surgery^4^. Chemotherapy can be employed as the initial treatment (first-line therapy) on its own or in conjunction with other treatment modalities^5^. Although conventional cancer therapies have seen significant progress in recent years, they also carry a significant risk of severe side effects because they target both healthy cells and tumor cells^6^. The non-specific nature of these therapeutic methods and the emergence of drug resistance during prolonged use necessitate the urgent development of novel treatment options^7^.

In recent years, there has been a significant push to design effective strategies for delivering chemotherapeutic molecules. The concept of targeted drug delivery has been around for years, with the goal of overcoming the limitations of non-specific drug distribution, unwanted side effects, and high dosages^8^. The development of truly effective tools for precise delivery of chemotherapeutic molecules to the target site is of paramount importance to overcome all biological barriers^9^. Emerging nanotechnology-based techniques in cancer therapy have demonstrated promising results. Therefore, the development of efficient, cost-effective, and highly biocompatible treatment alternatives is essential^4^. The development of nanoparticles smaller than 1 micrometer has propelled nanotechnology to become one of the world’s fastest-growing fields^10^. Their small size endows them with a high surface area-to-volume ratio, enhancing drug efficacy, surface contact, solubility, and reactivity, and expanding their potential applications^11^.

A wide spectrum of organic and inorganic nanoparticles, particularly metal nanoparticles (NPs) and their composites, has been extensively studied and employed in diverse applications^12,13^. The development of NP-based drugs has proven effective in minimizing side effects and achieving targeted action on cancer cells due to their large surface area. Notably, inorganic NPs, including metal and metal oxide particles such as gold, nickel, silver, iron oxide, zinc oxide, gadolinium, and titanium dioxide, hold promise for various medicinal applications, including cell imaging, biosensing, drug/gene delivery, and cancer therapy^14^. While nanoparticles can themselves act as toxic agents, zinc oxide nanoparticles (ZnO NPs) stand out as a promising candidate due to their inherent selective cytotoxicity towards cancer cells^15^.

Zinc oxide nanoparticles (ZnO NPs) have garnered significant attention due to their advantageous properties, including biocompatibility, environmental friendliness, low cost, ease of fabrication, high photosensitivity, large excitation binding energy, high thermal conductivity, and stability under harsh environmental conditions^16^. ZnO NPs possess the ability to absorb ultraviolet rays while remaining transparent to visible light, making them excellent sunscreen agents^17^. Numerous research studies have shown that ZnO NPs exhibit selective cytotoxicity towards cancer cells, effectively targeting and destroying cancerous cells through selective localization and cell cytotoxicity mechanisms^18^. The anticancer and antibacterial properties of ZnO NPs stem from their ability to induce the generation of reactive oxygen species (ROS). ZnO NPs possess a remarkable ability to trigger oxidative stress in cancer cells, which has been identified as a key mechanism of their cytotoxicity. The semiconductor nature of zinc oxide is responsible for its ability to generate ROS, leading to oxidative stress and eventually cell death when the cell’s antioxidant capacity is surpassed. Furthermore, the abundance of -OH groups on the surface of ZnO enables its slow dissolution in both acidic (such as tumor cells and the tumor microenvironment) and strongly basic conditions. ZnO NPs have attracted significant attention in biomedical applications due to their ability to inhibit angiogenesis, platelet aggregation, and inflammation, as well as their potential as dental materials and anticancer agents^1^.

Similar to other metal oxide NPs, ZnO NPs have been the subject of numerous studies investigating their potential toxicity. These studies have demonstrated that ZnO NPs can exhibit toxic effects on various cell lines and animals through a variety of mechanisms^19^. The low toxicity of zinc under physiological conditions (pH = 7.4) can be attributed to its limited release of Zn^2+^ ions. Comparable to elemental zinc, ZnO NPs exhibit biocompatibility towards normal mammalian cells due to their slow dissolution rate. However, at slightly acidic pH, ZnO NPs rapidly dissolve into Zn^2+^ ions, leading to oxidative stress and subsequent cell damage in cancer cells, demonstrating pH-responsive cytotoxicity^20^. Additionally, it is important to note that ZnO nanoparticles have a natural tendency to aggregate in various environments, including biological media^21^. This aggregation can influence their stability and functionality by reducing the available surface area and altering the distribution of nanoparticles within biological systems^22^. Such aggregation is often observed in suspensions and can be exacerbated in biological media due to the presence of proteins and other biomolecules that can induce or stabilize aggregates. This aggregation behavior necessitates careful consideration of nanoparticle modification strategies, such as coating with biocompatible materials, to enhance their dispersion, stability, and overall functionality in biological applications.

A wide range of approaches have been explored to achieve targeted delivery of anticancer drugs and minimize the toxicity of nanoparticles at specific concentrations. One promising strategy involves the utilization of functional ligands that can selectively bind to specific receptors that are overexpressed on cancer cells. In addition, cell-penetrating peptides or ligands for tight junction opening in tumors are being actively pursued as strategies to enhance the intracellular delivery of anticancer drugs. These functional ligands can be used alone or in combination to improve the intracellular uptake and target selectivity of anticancer drugs^23,24^. Moreover, the optoelectronic properties of ZnO can be affected by adsorbed proteins. Bovine serum albumin (BSA), as a model protein, is widely used in biomedical applications for its low toxicity and compatibility with biological systems^25,26^. Its coating improves the stability of ZnO NPs by preventing agglomeration and enhances their hydrophilicity, which is crucial for maintaining a uniform dispersion in aqueous solutions. Additionally, BSA’s functional groups facilitate interactions with cellular components, potentially improving the nanoparticles’ uptake and targeting efficiency. Various studies have also shown that the fluorescence intensity of BSA decreases when ZnO NPs are present, suggesting that energy transfer occurs from excited BSA to ZnO in the ZnO–BSA complex^27^.

Coating nanoparticles with inorganic compounds not only ensures their stability but also substantially broadens their applicability. Silicon compounds are the most commonly employed for this purpose. Silica, a classic coating material for nanoparticles, enhances their dispersion in aqueous solutions, increases their durability, and provides protection in acidic environments. Encapsulating nanoparticles with a silica (SiO_2_) shell using tetraethoxysilane (TEOS) as a silicon precursor effectively mitigates their potential toxicity and enhances their colloidal stability within the physiological pH range^28^. Therefore, coating the ZnO NPs with BSA and TEOS can increase their selective ability as anticancer drugs and reduce their toxicity at higher concentrations.

While previous studies have explored the anticancer potential of ZnO NPs, this research introduces a novel approach by synthesizing and characterizing ZnO NPs coated with BSA and TEOS. The unique combination of these coatings aims to enhance the stability, dispersibility, and selective cytotoxicity of ZnO NPs, particularly against colorectal cancer cells (Caco-2). Unlike previous studies that primarily focused on unmodified ZnO NPs, this study investigates the synergistic effects of dual-coating with BSA and TEOS, which not only improves the nanoparticles’ biocompatibility but also enhances their cellular uptake and targeted delivery capabilities. Furthermore, this research employs a comprehensive suite of biophysical techniques, including UV-Vis spectroscopy, DLS, FTIR, XRD, and TEM, to thoroughly characterize the modified nanoparticles. The study also evaluates the differential uptake and cytotoxic effects of these nanoparticles on both cancerous (Caco-2) and non-cancerous (HepG2) cell lines, providing a deeper understanding of their selective anticancer activity^29–31^. By demonstrating the enhanced stability, reduced toxicity, and improved targeting efficiency of BSA- and TEOS-coated ZnO NPs, this research offers a promising alternative to conventional chemotherapy, paving the way for more effective and safer cancer treatments.

## Experimental details

### Materials

Fluorescein isothiocyanate (FITC), acridine orange (AO), ethidium bromide (EB), puriss sodium hydroxide pellets, 2-propanol ACS reagent 99.5%, tetraethyl orthosilicate reagent grade 98%, and bovine serum albumin 98% lyophilized powder were obtained from Sigma-Aldrich (USA). Zinc acetate dihydrate 99.5% p.a. ACS was obtained from Roth (Germany). DMEM medium, fetal bovine serum (FBS), Ultraglutamine, penicillin/streptomycin, amphotericin B, and trypsin-EDTA were obtained from Lonza (Belgium). A WST-1 Cell Proliferation Assay Kit was obtained from Takara (Japan). The Alexa Fluor 488 Annexin V/Dead Cell Apoptosis Kit with Alexa Fluor 488 Annexin V and PI were obtained from Molecular Probes, Life Technologies (USA). Glass-based Petri dishes were obtained from SPL Life Science (Korea). Visking dialysis tubes were obtained from Serva Electrophoresis (Germany). Propidium iodide (PI), Rhodamine 123, Triton X-100, and dimethyl sulfoxide (DMSO) were obtained from Sigma-Aldrich (USA). Flow cytometry tubes were obtained from BD Biosciences (USA). All other reagents for flow cytometry, including buffers, were sourced from Cell Signaling Technology (USA). All chemicals and reagents were of analytical grade or higher. The chemicals were used as received.

### Synthesis of the ZnO nanoparticles

ZnO nanoparticles (ZnO NPs) were synthesized as a colloidal suspension prepared following a previously described method^32–35^. Briefly, 8.7796 g of zinc acetate dihydrate (99.5%) was dissolved in 80 ml of 2-propanol (99.9%) at 50 °C and later diluted to 920 ml. Next, 80 ml of a 1.6 g sodium hydroxide (99.5%) solution, prepared in a 7:1 mixture of 2-propanol and deionized water, was added at 0 °C to slow reaction kinetics, allowing controlled nucleation and growth, which results in smaller, uniform nanoparticles and prevents agglomeration, leading to higher purity and more consistent particle size. This addition occurred over 1 min, followed by continuous stirring for 30 min. The mixture was immersed for 2 h in a water bath preheated to 65 °C. After three days of further aging at room temperature, the solvent was removed by centrifugation at 7000 rpm for 15 min. The centrifugation process was repeated until the residual chemicals were completely removed. The precipitate was dried in an oven at 120 °C for 3 h. Afterward, the powder was calcined in a muffle furnace at 400 °C for 2 h^32^. Then, characterization measurements were performed using a UV-Vis spectrophotometer along with DLS, ELS, XRD, and TEM.

### Preparation of TEOS and BSA capped ZnO NPs

Zinc oxide (ZnO) was dispersed in water or capped with a selection of ligands using the following protocol, which was modified from a previous report^36^. ZnO NPs (100 mg) were sonicated for 15 min at room temperature for tetraethyl orthosilicate (TEOS), or at 4 °C for bovine serum albumin (BSA), in an ultrasonic bath along with 5 ml of 2-propanol. Next, 100 ml of a 2% solution of TEOS in DI water, or 100 ml of an 18.8 µM solution of BSA (1.25 mg/ ml) in DI water, was introduced, and the suspension was sonicated for 1 h at room temperature (TEOS), or with a gradual increase in temperature to room temperature (BSA). The lower concentration of BSA compared to TEOS in ZnO NPs modification is due to differences in their molecular structure and binding mechanisms. BSA, a large protein, covers a significant surface area with small amounts and forms a stable protein Layer on ZnO nanoparticles through multiple binding sites. On the other hand, TEOS, a smaller molecule, requires a higher concentration to form an effective silane layer. The NPs were then reacted overnight at room temperature (plain and BSA) or at 60 °C (TEOS) with shaking before being purified from the excess ligand and/or organic solvent residue by triple centrifugation/decantation (13,000 rpm, 15 min, room temperature). The capped ZnOB and ZnOT NPs were then redispersed by sonication in ultrapure water at a concentration of 1 mg/ml.

### Fluorescein isothiocyanate (FITC) labeling of ZnO, ZnOB and ZnOT

The NPs were labeled using the procedure described as follows: 25 mg of ZnO, ZnOB, and ZnOT nanoparticles were suspended in 50 ml of deionized water and left stirring for 30 min or until completely suspended. The pH of the solution was adjusted to 6.5 using 1 M HCl, after which 2.5 mg of FITC dissolved in DMSO (1 mg/ml) was added to the solution. The solution was then stirred overnight in the dark. Afterward, the solution was centrifuged at 5000 rpm at 4 °C for 20 min, the supernatant was discarded, and centrifugation was repeated several times with deionized water until no FITC remained in the supernatant. To verify successful FITC conjugation, absorbance from 400 to 700 nm was measured using a spectrophotometer. After removing the residual unreacted FITC, the ZnO-FITC (ZnOF), ZnOB-FITC (ZnOBF), and ZnOT-FITC (ZnOTF) pellets were suspended in deionized water.

### Structural characterization of the zinc oxide nanoparticles

The ZnO, ZnOF, ZnOB, ZnOBF, ZnOT and ZnOTF nanomaterials were characterized using a UV-Vis-NIR spectrophotometer (Cary 5000 UV-Vis-NIR; Varian, U.K.). The zeta potentials and particle sizes of the nanomaterials were determined by laser diffraction spectrometry (Malvern, U.K.) and high-resolution transmission electron microscopy (HRTEM, Tecnai G20, FEI, Netherlands) operating at 200 kV. The morphologies of the nanomaterials were characterized using TEM. Nanoparticle samples were stained with phosphotungstic acid, and the TEM images were obtained under negative contrast. Fourier transform infrared spectroscopy (FTIR) analysis was conducted using a JASCO FTIR 6100 spectrometer (Japan). Identification of the peaks and bands was performed using IRPAL 2.0 (Table driven Infrared Application) software. The characterization procedures and experiments were performed at the Nanotechnology and Advanced Materials Central Lab.

### Biological characterization of the zinc oxide nanoparticles

#### Cell culture

Human colorectal (Caco-2) cells, hepatocyte (HepG2) cells, and human embryonic kidney (HEK293) cells were obtained from the cell culture laboratory at VACSERA, Egypt. The cells were maintained in Dulbecco’s modified Eagle’s medium (DMEM) supplemented with 100 µg/ml of streptomycin, 100 units/ml of penicillin, and 10% fetal bovine serum (FBS), in a humidified atmosphere containing 5% CO_2_ at 37 °C for 24 h to form a confluent monolayer and permit cell adhesion. All cell culture supplies were obtained from Lonza (Germany).

#### Cytotoxicity evaluation of the zinc oxide nanoparticles

The HEK293, HepG2, and Caco-2 cells were used for cytotoxicity evaluation. They were seeded into 96-well plates at a density of 10 × 10^3^ cells/well using 200 µl of growth media (10% FBS, 1% alanyl-L-glutamine, 1% penicillin, streptomycin, and 0.1% amphotericin) in each well and were incubated for 24 h. Next, the growth media was replaced with 200 µl of growth media containing ZnO, ZnOT, or ZnOB nanoparticles at concentrations of up to 400 µg/ml for each nanoparticle formulation. After incubating for 24 h, the media was replaced with 100 µl of fresh media and 10 µl of a water-soluble tetrazolium salt (WST-1) solution (Takara, Japan) to assess cell viability. After an additional incubation period of 2 h, the OD was determined at 450 nm and a reference absorbance was measured at 630 nm using a Sunrise microplate spectrophotometer (Tecan, USA)^37–39^. The experiments were conducted in triplicate and repeated twice. The results are expressed as the mean ± standard deviation.

#### Live/dead cell assay by CLSM

Cells were seeded into eight glass Petri dishes at a density of 10 × 10^4^ cells/dish and were cultured for 24 h in 2 ml of growth media. Next, the growth media was replaced with media containing ZnO, ZnOB, or ZnOT at their IC_50_ concentrations. After incubating for 24 h, 10 µl of an AO and EB mixture at a concentration of 100 µg/ml for each dye was added. The Petri dishes were incubated at 37 °C for 15 min. Next, the cells were observed by CLSM^37,40^.

#### Apoptosis by CLSM and flow cytometry

The ability of zinc oxide nanoparticles to induce apoptosis was performed using Alexa Fluor^®^ 488 Annexin V – Propidium iodide (PI) assay. In this assay, viable cells have a uniform bright green nucleus with a normal structure^10,37,41^. ZnO, ZnOB, or ZnOT nanoparticles were incubated with cells for 24 h under normal incubation conditions at their IC_50_ concentrations with respect to the effect of each nanomaterial. In the glass Petri dish, 100 µl of a 1× annexin binding buffer was added to 5 µl of Alexa Fluor^®^ 488 Annexin V and 1 µl of a 100 µg/ml PI working solution. The cells were then incubated at room temperature for 15 min. Afterward, 400 µl of the 1× Annexin binding buffer was added, followed by gentle mixing and maintaining the Petri dishes on ice. The cells were then viewed under CLSM at 488 nm and 543 nm for Alexa Fluor 488 and PI, respectively, according to the manufacturer’s instructions. All imaging studies were performed using an LSM 710 (Carl Zeiss, Germany) with an image size of 1024 × 1024 pixels, a 16-bit depth, and Plan-Apochromat 63×/1.4 oil DIC and EC Plan-Neofluor 40×/1.3 oil DIC objectives. The acquisition was performed using Zen 2009 software, and processing was conducted using Zen 2012 (blue and black edition).

To evaluate apoptosis induction in treated cells, HEK293, HepG2, and Caco-2 cells using flow cytometry. Post-treatment, both floating and adherent cells were collected by trypsinization, washed with cold PBS, and resuspended in 100 µl of 1X Annexin V binding buffer. Cells were then stained with 5 µl of Alexa Fluor^®^ 488 Annexin V and incubated for 20 min at room temperature. After incubation, 400 µl of binding buffer was added to each sample then 5 µl of propidium iodide (PI, 50 µg/ml) was added and the samples were analyzed immediately by CytoFlex V5-B5-R3 flow cytometer using the 488 nm laser. 10,000 events were recorded from each sample and the data was analyzed using CytExpert 2.3 software. A doublet discrimination chart was used to exclude aggregates and duplicates from the downstream analysis The applied gating strategy was: live cells “Annexin V^−^, PI^−^”, early apoptotic cells “Annexin V^+^, PI^−^”, late apoptotic cells “Annexin V^+^, PI^+^” and necrotic cells “Annexin V^−^, PI^+^”.

#### Cell cycle analysis

To assess the effects of ZnO, ZnOB, and ZnOT nanoparticles on cell cycle progression, HEK293, HepG2, and Caco-2 cells were seeded in 6-well plates (2 × 10⁵ cells per well) and allowed to adhere overnight. The cells were treated with ZnO, ZnOB, and ZnOT nanoparticles at 100 µg/ml concentration for 24 h. After treatment, both floating and adherent cells were collected, washed with cold PBS, and then fixed in 3 ml of 70% ice-cold, ethanol was added dropwise to the cell pellet while vertexing. Cells then were stored at 4 °C overnight. The fixed cells were washed twice with 5 ml cold PBS at 650 x g for 10 min, then resuspended in 500 µl of PI/RNase Staining Solution, and incubated at 37 °C CO_2_ incubator for 15 min then the cells emerged directly in ice till acquisition. The stained cells were analyzed by CytoFlex V5-B5-R3 flow cytometer using a 488 nm laser. 10,000 events were recorded from each sample and analyzed using CytExpert 2.3 software. The cell cycle stages (G0/G1-S-G2/M) were determined based on the control samples and the gates were then applied to the experimental samples, a doublet discrimination chart was used to exclude aggregates and duplicates from the downstream analysis. Histogram charts were created with the PI on the X-axis and cell count on the Y-axis to determine the number of cells per cell cycle phase.

#### Cellular uptake and internalization of the ZnO NPs by CLSM and flow cytometry

The Caco-2 cells were used to investigate the cellular uptake and internalization of the prepared nanoparticles^10,41^. They were cultured in glass Petri dishes at a density of 10 × 10^4^ cells/dish for 24 h in 2 ml of growth media. Next, the media was discarded and replaced with 2 ml of growth media containing ZnOF (10 µg/ml), ZnOBF (10 µg/ml), or ZnOTF (10 µg/ml) nanoparticles. The cells were incubated for 4 h at 4°C and at 37 °C and 5% CO_2_ in a humidified incubator for 4 and 24 h. At the designated time points, the media was discarded and the cells were washed three times with PBS followed by immediate CLSM imaging using a 488 nm excitation wavelength to assess the uptake of the FITC conjugated nanoparticles.

To evaluate the cellular uptake of ZnO, ZnOB, and ZnOT nanoparticle by flow cytometry, post-incubation, cells were washed with cold PBS, trypsinized, and resuspended in PBS for immediate analysis by flow cytometry using a 488 nm laser and a 530/30 nm bandpass filter for FITC detection. 10,000 events were recorded from each sample, and the data was analyzed using CytExpert 2.3 software. A doublet discrimination chart was used to exclude aggregates and duplicates from the downstream analysis. The mean fluorescence intensity (MFI) was calculated by the software to quantify the internalization of FITC-labeled nanoparticles by the cells.

#### Mitochondrial function assay by flow cytometry

Rhodamine 123 (Rh123), a fluorescent dye, was used to study mitochondrial membrane potential in HEK293, HepG2, and Caco-2 cells after treatment with ZnO, ZnOB, and ZnOT nanoparticles at 100 µg/ml concentration for 24 h^32^. The cells were labeled with Rh123 by incubating them with the dye (1 µg/ml) for 2 h at 37 °C and 5% CO_2_ in the incubator. After incubation, cells were washed first with cold PBS, trypsinized, and centrifuged then resuspended in 300 µl of PBS. The samples were analyzed immediately by CytoFlex V5-B5-R3 flow cytometer using a 560 nm laser to detect Rh123^+^ (live), Rh123^dim^ (stressed), and Rh123^−^ cells (dead). 10,000 events were recorded from each sample and the data was analyzed analysis using CytExpert 2.3 software. Data analysis focused on a doublet discrimination chart to exclude aggregates and duplicates from the downstream analysis, and then mean fluorescence intensity (MFI) of Rh123 was calculated.

### Statistical analysis

All data were analyzed through Origin 8.0 (Origin Lab Corporation, USA) and shown as the average ± standard deviation. All experiments were performed in triplicate (*n* = 3). The significance was calculated by Student’s ANOVA test using GraphPad Prism 7.00, where values of *p* < 0.05 were considered statistically significant. Graphs are marked as follows: * significant at *p* < 0.05, ** significant at *p* < 0.01, *** significant at *p* < 0.001, and **** significant at *p* < 0.0001.

## Results and discussion

### Characterization of the ZnO, ZnOB, and ZnOT nanoparticles

**Fig. 1:**
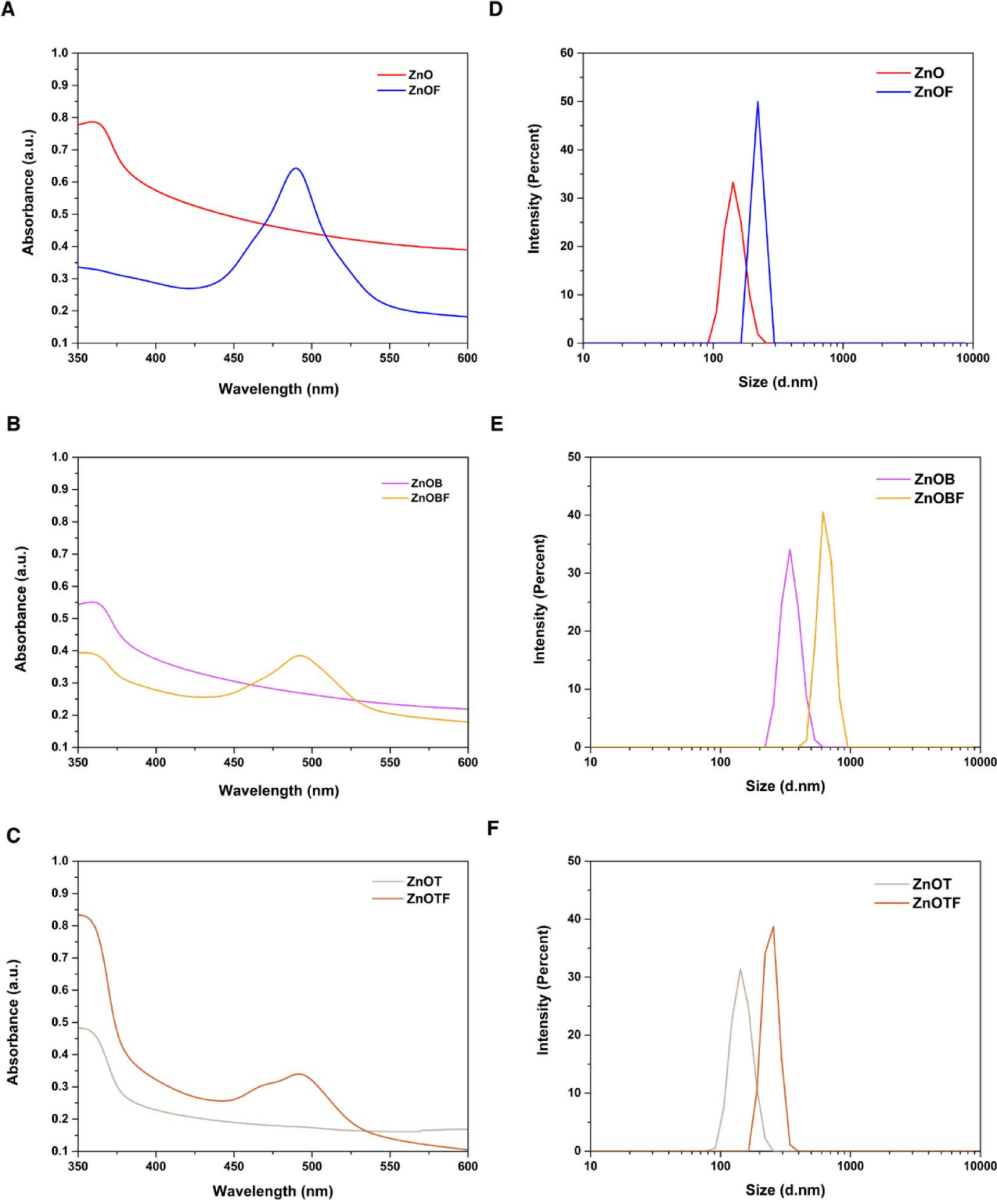
UV-Vis absorption spectra and DLS analysis of ZnO nanoparticle formulations. (**a**–**c**) UV-Vis absorption spectra of ZnO, ZnOB, and ZnOT nanoparticles and their respective FITC-labeled forms, showing spectral shifts associated with size and surface modifications. (**d**–**f**) DLS particle size distribution of ZnO, ZnOB, and ZnOT nanoparticles and their FITC-labeled forms, illustrating changes in hydrodynamic diameter and monodispersity upon surface functionalization.

Absorption spectroscopy is a powerful nondestructive method for evaluating the optical properties of applicable nanoparticles. Additionally, the UV–Vis absorption spectrum is a function of the nanoparticles (NPs) size as it has a direct effect on λ_max_. The maximum wavelength(λ_max_) exhibits an inverse relationship with quantum confinement effects in smaller particles, leading to a blue shift in smaller sizes and a red shift in larger ones. In other words, there is a direct relationship between the absorption peak of the ZnO NPs and their size. Based on this fact, a larger λ max for the NPs and lower energies leads to a redshift in the absorbance spectrum of the ZnO NPs. Therefore, the absorption spectra of the different forms of the ZnO NPs were measured and represented in Fig. 1. The measured λ_max_ values of 363 nm for ZnO, 361 nm for ZnOB, and 357 nm for ZnOT indicate a slight shift in ZnOB and ZnOT due to changes in particle size compared to ZnO, confirming the relationship between λ_max_ and particle size. A generic excitation absorption at 492 nm was observed for the FITC-labeled nanoparticles (ZnOF, ZnOBF, and ZnOTF). From these λ_max_ values, the particles size of the different forms of ZnO were expected to be consistent after modification with BSA or TEOS, which enhanced the dispersibility and stability of the ZnO NPs in an aqueous solution.

The shift observed in the UV–Vis spectra of ZnOT and ZnOB nanoparticles after modification is attributed to potential electronic interactions between the nanoparticle surfaces and the modifiers. These interactions likely result in charge transfer or changes in surface electronic density, stabilizing the nanoparticles and leading to lower energy transitions. Furthermore, the stabilizing effect of BSA and TEOS enhances nanoparticles dispersion and reduces aggregation.

Particle size plays a key role in the cellular uptake of NPs and is a critical parameter for cationic polymers intended for application use^42,43^. Therefore, the sizes of the nanoparticles were measured using dynamic light scattering (DLS). The average particle sizes and polydispersity index (PDI) values for the nanoparticles are as follows: ZnO exhibited a size of 140 ± 13.6 nm with a PDI of 0.457, indicating moderate uniformity. ZnOB and ZnOT nanoparticles had average particle sizes of 342 ± 8.4 nm and 145 ± 23.8 nm, with PDI values of 0.307 and 0.398, respectively, showing relatively higher monodispersity. This trend aligns with the optical absorption properties, where larger particle sizes (ZnOB) correlate with a red shift in λ_max_.

The size of particles and their Polydispersity Index (PDI) play a crucial role in how well cells absorb them and how effective they are in treatment. These factors can significantly impact both the cellular uptake and the overall therapeutic success of a treatment. Nanoparticles such as ZnOT, are generally more efficiently internalized by cells via endocytosis due to their optimal size for membrane interactions and cellular transport. Larger particles, such as ZnOB, can exhibit enhanced retention at the target site due to slower clearance rates, potentially increasing localized therapeutic effects. Additionally, lower PDI values indicate higher monodispersity, which ensures uniform interaction with biological systems and reduces variability in cellular uptake. These characteristics are particularly critical for applications in drug delivery, where controlled and consistent uptake enhances the therapeutic index of the nanoparticles. For the FITC-labeled variants, ZnOF, ZnOBF, and ZnOTF, particle sizes were 220 ± 11.05 nm, 615 ± 16.21 nm, and 255 ± 31.5 nm, with PDI values of 0.537, 0.459, and 0.475, respectively. The PDI values confirm a consistent particles size distribution across formulations, with slight increases in labeled variants due to the labeling process, which did not significantly impact nanoparticle behavior in evaluation tests (Fig. 1).

**Fig. 2:**
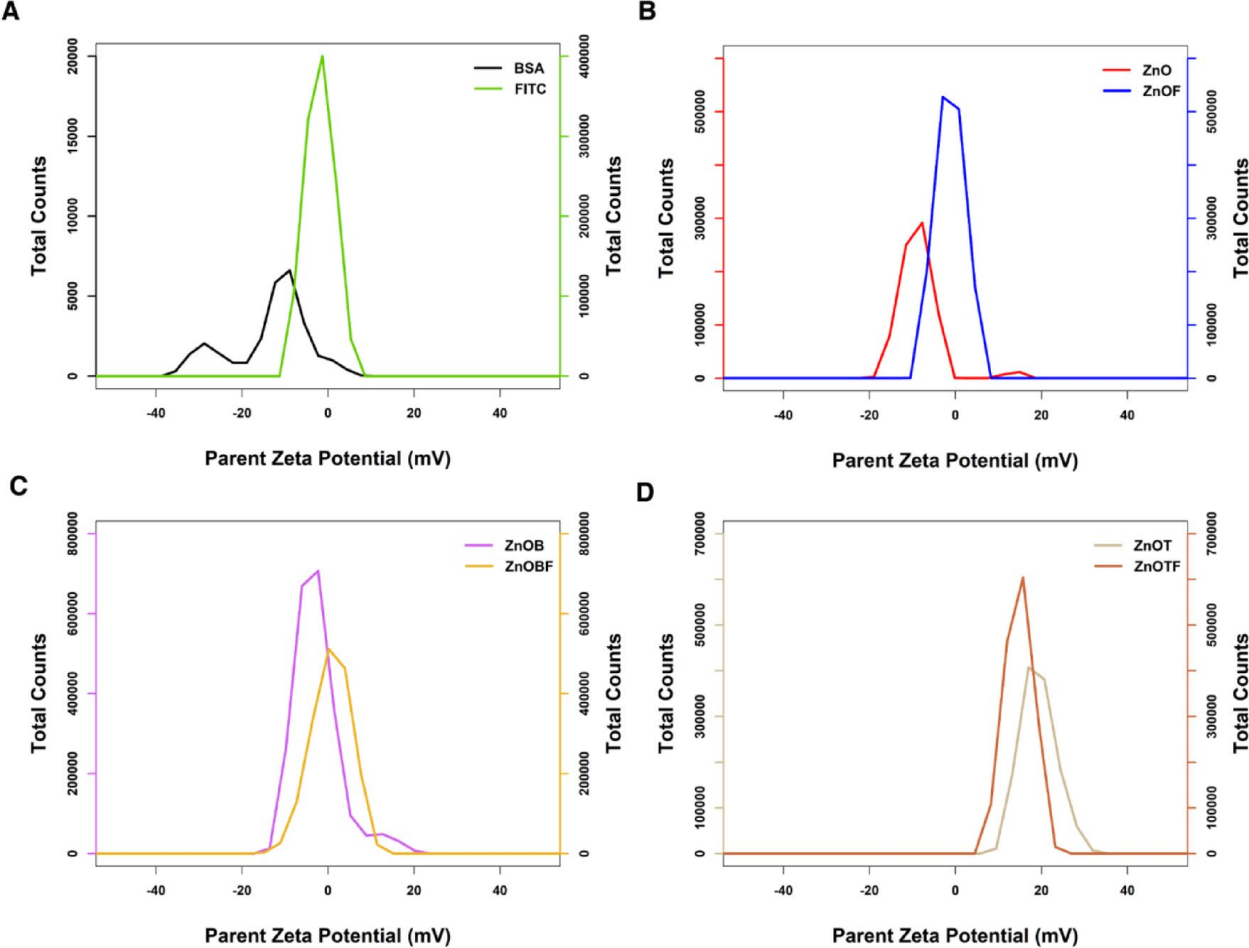
Zeta potential of modified and labeled ZnO nanoparticles. (**a**) Zeta potential of control molecules (FITC and BSA). (**b**–**d**) Zeta potentials of ZnO, ZnOB, and ZnOT nanoparticles with and without FITC labeling, showing surface charge variations due to functionalization and their implications for stability and biological interactions.

The zeta potential of NPs determines their colloidal stability and influences the effectiveness of their interactions with the cell membrane. Thus, the zeta potentials of NPs can affect their efficiencies^42^. The measured zeta potentials were − 19.21 ± 3.38, -11.24 ± 3.31, -3.24 ± 4.3, 3.45 ± 4.64, 19.3 ± 4.16, and 14.7 ± 3.29 mV for the ZnO, ZnOF, ZnOB, ZnOBF, ZnOT, and ZnOTF nanoparticles, respectively, as illustrated in Fig. 2. The surface charge of nanoparticles, as indicated by the zeta potential, plays a pivotal role in their interaction with biological systems. Negatively charged nanoparticles, such as BSA-coated ZnO NPs, tend to interact less aggressively with negatively charged cell membranes due to electrostatic repulsion, potentially reducing cytotoxicity and improving biocompatibility. In contrast, positively charged nanoparticles, like ZnOT and ZnOTF, exhibit stronger electrostatic interactions with cell membranes, which can enhance cellular uptake but may also increase the risk of membrane disruption and toxicity.

FITC-modified ZnO nanoparticles exhibit zeta potential values closer to neutrality, suggesting a reduction in electrostatic interactions. This neutral charge distribution may facilitate penetration into tissues with dense extracellular matrices or limit nonspecific interactions, improving biodistribution and reducing off-target effects. In contrast, BSA presents multiple peaks, suggesting higher variability in its zeta potential, with a maximum peak showing a negative value (− 10.6 ± 4.92) lower than that of FITC (− 1.92 ± 3.34). This indicates that BSA-coated nanoparticles likely possess a more negative surface charge compared to those labeled with FITC, which aligns with the protein’s nature, as it contains carboxylic and amide groups. The ZnO NPs demonstrated poor stability in suspension, attributed to their tendency to aggregate, a conclusion supported by the zeta potentials of the various ZnO forms. Modifying ZnO with TEOS resulted in a positive zeta potential, potentially altering its interactions with cells. The interaction between ZnO NPs and FITC likely relies on both electrostatic and van der Waals forces, as evidenced by the change in zeta potential upon adding FITC to the nanoparticles, which approaches the isoelectric point regardless of their initial zeta potential. The isoelectric point (IEP) is the pH at which a nanoparticle’s net surface charge becomes neutral. At this point, the electrostatic repulsion between particles is minimized, increasing the likelihood of aggregation. In biological environments, the IEP also governs how nanoparticles interact with biomolecules, such as proteins and cell membranes. For example, nanoparticles at or near their IEP may show reduced stability in suspension, impacting their biodistribution and clearance rates. Conversely, a zeta potential far from the IEP suggests enhanced colloidal stability, which is essential for maintaining consistent nanoparticle behavior in vivo. The shift toward the IEP upon FITC labeling highlights the balance between stabilizing forces and functionalization-induced changes, which could influence the nanoparticles’ biological interactions and therapeutic efficacy. Overall, the observed zeta potential variations highlight the versatility of surface modifications in tailoring nanoparticles for specific therapeutic applications. For instance, the positively charged ZnOT nanoparticles could be used for localized drug delivery to cancerous tissues, where enhanced cellular uptake is desirable, while the negatively charged ZnOB nanoparticles could be employed for systemic applications requiring lower nonspecific interactions.

**Fig. 3:**
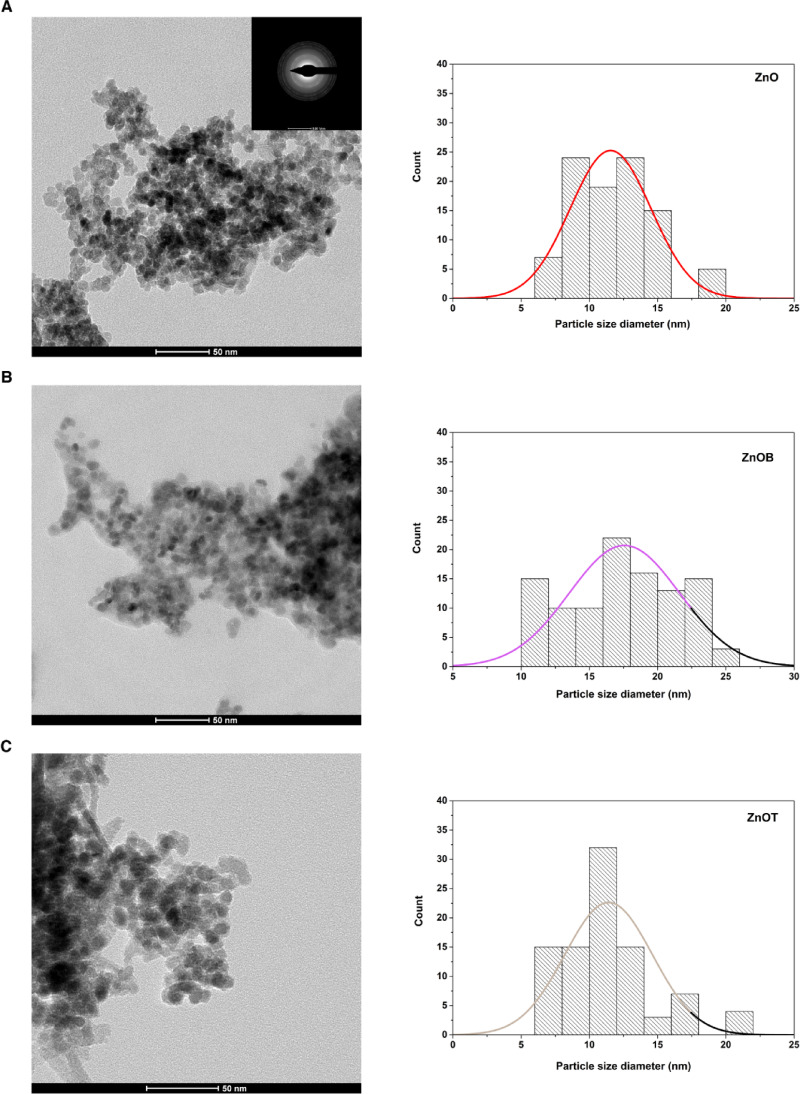
TEM images and size distributions of ZnO, ZnOB, and ZnOT nanoparticles. TEM micrographs confirm the spherical morphology, size uniformity, and agglomeration behavior of nanoparticles. The presence of BSA and TEOS shells around ZnOB and ZnOT respectively, are also observed, with consistent size ranges compared to DLS data.

The morphologies and size distributions of ZnO, ZnOB, and ZnOT nanoparticles were further confirmed by TEM analysis (Fig. 3). The electron diffraction pattern of ZnO revealed the crystalline nature of the zinc oxide nanoparticles (Fig. 3). The average diameters of ZnO, ZnOB, and ZnOT as measured from TEM images were 14 ± 5.2 nm, 19 ± 6.8 nm, and 17.8 ± 7.4 nm, respectively. Agglomeration was observed in all the nanoparticle formulations, this is a typical characteristic of ZnO NPs^44^. The agglomeration observed in TEM images has potential implications for biological applications and in vivo behavior. Agglomeration can influence cellular uptake by reducing the effective surface area and altering the interaction of nanoparticles with cell membranes. While smaller, well-dispersed nanoparticles are often preferred for efficient cellular internalization, agglomerates may exhibit slower uptake rates but can benefit from prolonged circulation times in vivo due to decreased renal clearance.

BSA and TEOS formed shells surrounding ZnO NPs with diameters of 3.6 and 5.6 nm for ZnOB and ZnOT, respectively. These shells play a critical role in enhancing the stability and functional properties of the nanoparticles. The BSA coating provides steric stabilization, reducing aggregation by forming a protein layer that acts as a physical barrier, which is particularly useful for maintaining colloidal stability in biological environments. Additionally, the functional groups present in BSA, such as carboxyl and amine groups, may enhance biocompatibility and facilitate conjugation with biomolecules for targeted delivery applications. Similarly, the TEOS-modified shell introduces a silica-like coating, known for its chemical inertness and ability to improve dispersibility. The increased shell thickness in ZnOT nanoparticles contributes to their colloidal stability and may provide additional protection against enzymatic degradation in biological systems. This stability ensures that the nanoparticles retain their structural integrity during cellular uptake and enhances their overall functional properties for biomedical applications.

A quantitative analysis of size distribution was conducted using multiple TEM images (Fig. 3), and the results demonstrated consistency with the DLS measurements. Although TEM tends to show smaller average sizes due to the drying and potential aggregation of particles on the grid, the observed agglomeration in all formulations is consistent with the DLS data. This confirms that the larger particle sizes observed in DLS are likely due to the formation of agglomerates in solution. The particle sizes observed in this study are in agreement with reported literature values for similar ZnO nanoparticle systems, where ZnO particles typically range from 10 to 50 nm for TEM measurements, depending on synthesis and stabilization methods^45,46^. The slightly larger diameters observed for ZnOB and ZnOT align with findings from studies involving surface modifications with BSA and TEOS, which contribute to increased shell thickness and enhanced dispersibility^47,48^. These comparisons contextualize our results within the broader framework of ZnO nanoparticle research and further validate the consistency between the DLS and TEM data. Overall, the TEM size distribution analysis provides complementary information, validating the DLS results and offering a comprehensive understanding of the particle size characteristics.

**Fig. 4:**
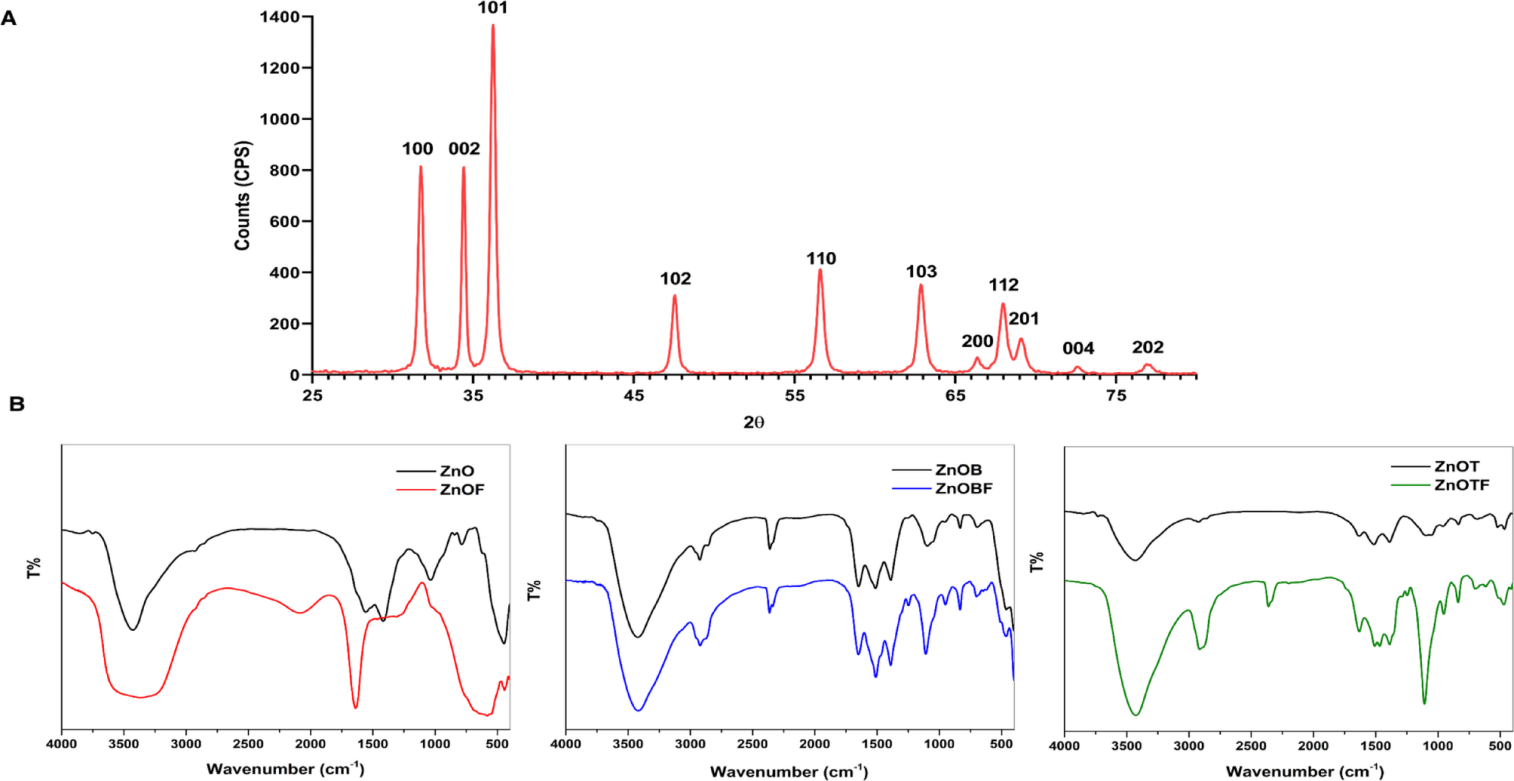
XRD and FTIR spectra of ZnO nanoparticles and their derivatives. (**a**) XRD patterns confirming the crystalline wurtzite structure of ZnO nanoparticles. (**b**) FTIR spectra demonstrating characteristic peaks corresponding to surface functional groups, coatings (BSA and TEOS), and FITC modifications, validating successful nanoparticle functionalization.

Figure 4 shows the XRD pattern of the ZnO NPs grown by using the hydrothermal synthetic method. The dominant diffraction peak for the (002) plane indicates a high degree of orientation^49^. This pattern demonstrated the hexagonal wurtzite structure of the ZnO NPs. The ZnO wurtzite structure has a hexagonal unit cell with two lattice parameters, a and c, and belongs to either the $\:{C}_{6v}^{4}\:$or P63 mc space group. In wurtzite hexagonal ZnO, each anion is surrounded by four cations at the corners of a tetrahedron, which reflects the tetrahedral coordination of the anions and hence exhibits sp^3^ covalent bonding^50^. The XRD pattern showed the orientation and crystal nature of the zinc oxide nanoparticles. The diffraction pattern of the ZnO NPs presented distinctive peaks for the (100), (002), (101), (102), (110), (103), (200), (112), (201), (004) and (202) planes for the peak positions with 2θ values of 31.79°, 34.48°, 36.32°, 47.55°, 56.69°, 62.96°, 66.43°, 67.96°, 69.17°, 72.57° and 77.05°, respectively. These results are in agreement with the standard peaks of the hexagonal phase structure of ZnO according to the ICDD database library (JCPDS File No. 00–001–1136). All peak positions and relative peak intensities of the ZnO product agreed well with those of the standard XRD pattern, indicating that the ZnO product was of high purity. Moreover, all the diffraction peaks of the product showed strong peak intensities, indicating that the obtained ZnO NPs had a high degree of crystallinity, which was in agreement with the results reported by L. Shen et al.^44^ and Y. Chung et al.^51^. Additionally, the crystallite sizes for each diffraction peak were calculated using the Scherrer equation, and the results are summarized in Table 1. The calculated crystallite sizes varied significantly across different peaks, ranging from 12.6 nm (126 Å) to 49.7 nm (497 Å). These results further confirm the nanoscale crystalline nature of the ZnO particles. Additionally, these size variations can have significant implications for the optical and mechanical properties of the nanoparticles.

The crystallite size influences the bandgap energy of ZnO nanoparticles. Smaller crystallites typically exhibit quantum confinement effects, leading to a wider bandgap and a corresponding blue shift in optical absorption spectra. Conversely, larger crystallites may result in reduced bandgap widening and are associated with higher light scattering. These effects are critical for applications such as UV-blocking coatings and optoelectronic devices. Crystallite size can also affect the mechanical properties of ZnO. Smaller crystallites generally contribute to enhanced hardness and strength due to the Hall–Petch effect, where grain boundaries act as barriers to dislocation motion. However, excessively small crystallites might lead to grain boundary weakening under certain conditions. Larger crystallites, on the other hand, may provide more flexibility but could reduce the material’s ability to resist mechanical deformation. These relationships underline the importance of tailoring crystallite size during synthesis to optimize ZnO nanoparticles for specific applications, such as optical sensors, antimicrobial agents, or structural composites.

Crystallite size also influences the reactivity and stability of nanoparticles, which are crucial for their therapeutic applications. Smaller crystallites, with their higher surface-to-volume ratio, have more active sites for chemical reactions, enhancing the efficacy of ZnO nanoparticles in drug delivery and antimicrobial applications. However, this also makes them more prone to oxidation and degradation, potentially reducing their stability in biological environments. Larger crystallites tend to be more stable due to fewer surface defects and a lower energy state. This increased stability can be beneficial in long-term drug delivery applications where sustained release is needed without rapid degradation of the nanoparticles. The stability of ZnO nanoparticles is also influenced by surface charge, agglomeration, and surface modifications (e.g., BSA or TEOS), which can alter their reactivity and behavior in vivo.

**Table 1:** Crystallite sizes of ZnO nanoparticles calculated using the scherrer equation based on XRD peak positions (2θ).

No.	Pos. [°2θ]	Crystallite Size [Å]
1	31.79	203
2	34.48	265
3	36.32	205
4	47.55	126
5	56.69	256
6	62.96	135
7	66.43	175
8	67.96	188
9	69.17	497
10	72.57	347
11	77.05	328

b) FTIR spectra of ZnO, ZnOB, ZnOT, and FITC-labeled nanoparticles, highlighting specific functional groups and chemical bonds formed during surface modifications. These spectra directly connect functionalization to enhanced biocompatibility and targeting capabilities.

FTIR spectra were obtained to investigate the interactions and the formed bonds of the ZnO, ZnOB, and ZnOT nanoparticles with FITC to estimate the possible peak shifts due to labeling the ZnO, ZnOB, and ZnOT nanoparticles with the FITC dye (Fig. 4). The peaks presented in the spectra of ZnO were in agreement with those reported in the literature^52,53^. A detailed summary of the functional groups and their corresponding peak positions is presented in Table 2. The FTIR spectra of ZnO, ZnOB, and ZnOT nanoparticles reveal significant structural changes due to the coating with FITC and modification with BSA. ZnO NPs exhibit characteristic peaks for FITC dye interaction at 2086 and 1024 cm^−1^, amide I groups at 1640 cm^−1^, OH vibrations between 400 and 800 cm^−1^, and NH_2_ stretching at 3370 cm^−1^^54–56^. Thiourea bond formation with FITC is indicated at 2086 cm^−1^. ZnOB NPs show phosphoramide P = O stretching at 1640 cm^−1^, confirming BSA attachment. They also display NH_2_ asymmetric stretching at 3370 cm^−1^, NH stretching at 3419 cm^−1^, and NH^3+^ stretching at 2164 cm^−1^, along with C–N and C-S symmetric stretching at 1108 and 701 cm^−1^, respectively. ZnOT NPs share similar BSA modification peaks and exhibit additional peaks due to thiourea bond formation and functional group interactions. Key shifts and increased intensities in peaks (e.g., 2915, 1632, 1509 cm^−1^) suggest further modifications compared to ZnOB. Ultimately, the spectra confirm the stabilization and coating of ZnO nanoparticles through various functional group interactions, including coordination with OH, NH, C–N, and aldehyde groups, as well as the formation of thiourea bonds with FITC and modifications from BSA.

The functional group interactions observed in the FTIR spectra significantly influence the nanoparticles’ physicochemical properties. For example, the incorporation of BSA improves colloidal stability by altering surface charge, as evidenced by zeta potential measurements. Thiourea bonds and other functional groups contribute to enhanced binding efficiency with targeting molecules such as FITC. The presence of functional groups such as NH_2_ and C–N contributes to improved interaction with biological molecules, while BSA coatings ensure reduced cytotoxicity and prolonged stability in physiological conditions. These features are vital for functional enhancements, such as targeted drug delivery, where surface functionality directly impacts cellular uptake and distribution.

**Table 2:** Summary of functional groups and their corresponding peak positions in FTIR spectra.

Functional Group	Peak Position (cm^−1^)	Assignment	Nanoparticles
OH stretching	3200–3600	Hydrogen-bonded OH groups	ZnO, ZnOB, ZnOT
C–N stretching	2800–2900	C–N stretching	ZnO, ZnOB, ZnOT
FITC stretching	2086, 1024	FITC dye interaction	ZnO
Amide I stretching	1640	Amide I groups	ZnO
OH vibrations	400–800	OH bending and stretching vibrations	ZnO
C–N symmetric stretching	1088, 1108	Symmetric stretching of C–N bond	ZnO, ZnOB, ZnOT
C-S symmetric stretching	701, 711	Symmetric stretching of C-S bond	ZnO, ZnOB
NCN asymmetric bending	579, 504	NCN asymmetric bending	ZnO, ZnOB
NH_2_ asymmetric stretching	3370	NH2 asymmetric stretching	ZnO, ZnOB, ZnOT
NH stretching	3162, 3260, 3419	NH stretching modes	ZnO, ZnOB, ZnOT
NH^3+^ stretching	2086, 2164	NH3 + stretching	ZnO, ZnOB
P = O stretching (Phosphoramide)	1640	P = O stretching in phosphoramide confirming BSA modification	ZnOB
Thiourea bond formation	2086	Thiourea bond formation with FITC	ZnO

### Cytotoxicity evaluation of the ZnO, ZnOB, and ZnOT nanoparticles

**Fig. 5:**
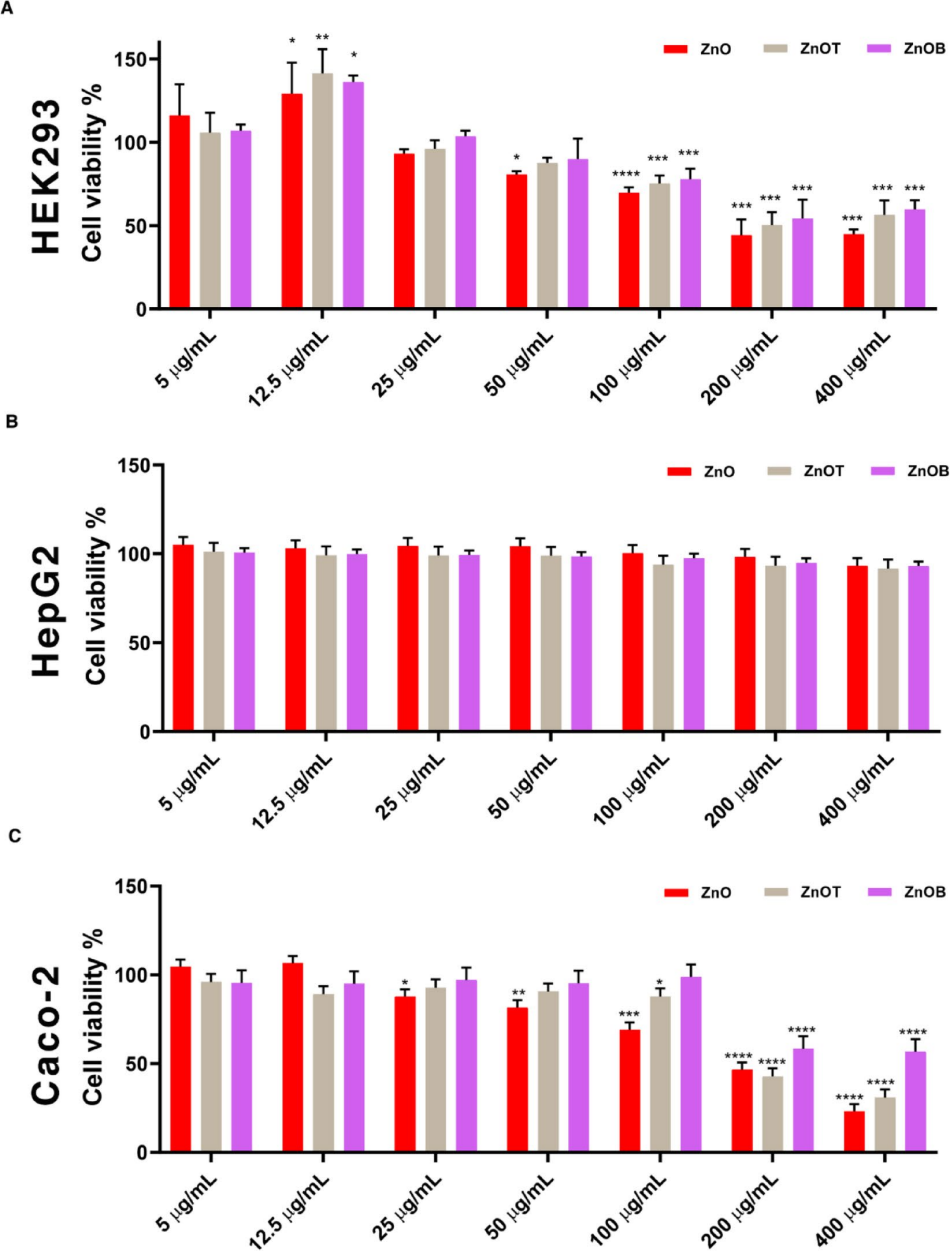
Cytotoxicity profiles of ZnO, ZnOB, and ZnOT nanoparticles across three cell lines. Cell viability results in HEK293 (**a**), HepG2 (**b**), and Caco-2 (**c**) cells after exposure to varying nanoparticle concentrations, highlighting dose-dependent cytotoxicity and selective anticancer activity toward Caco-2 cells.

The impact of ZnO, ZnOT, and ZnOB nanoparticles on cell viability was evaluated across different cell lines. For HEK293 cells (Fig. 5a), ZnOT and ZnOB demonstrated higher cell viability than ZnO at lower concentrations (5, 12.5, and 25 µg/ml), with ZnOT showing a statistically significant increase at 12.5 µg/ml (*p < 0.05, **p < 0.01). However, at higher concentrations (50 µg/ml and above), all nanoparticles caused a significant reduction in viability, with the most pronounced decrease at 100 µg/ml and beyond (****p < 0.0001). ZnOT and ZnOB appear to be less toxic at lower doses but exhibit increased cytotoxicity at higher concentrations, while ZnO consistently reduces viability with increasing concentration. This concentration-dependent cytotoxicity highlights the therapeutic efficiency of ZnOT and ZnOB for targeted therapy applications, minimizing off-target effects while ensuring efficacy at higher doses.

For HepG2 cells (Fig. 5b), cell viability remained close to 100% across all tested concentrations of the nanoparticles, showing no statistically significant changes. This suggests that HepG2 cells are resistant to the cytotoxic effects of ZnO, ZnOB, and ZnOT, indicating their biocompatibility. The observed resistance may result from reduced ROS generation, efficient antioxidant mechanisms, and lower nanoparticle uptake, highlighting their suitability for non-cancerous applications. In contrast, Caco-2 cells (Fig. 5c) maintained high viability at lower concentrations (5, 12.5, and 25 µg/ml) but showed a noticeable decline starting from 50 µg/ml, with ZnO exhibiting the highest cytotoxicity at concentrations of 100 µg/ml and above. The nanoparticles exhibit dose-dependent cytotoxicity, with ZnO being the most toxic. These results suggest that ZnO, ZnOT, and ZnOB have potential for targeted therapy in colorectal cancer due to their selective cytotoxicity at higher concentrations, while their biocompatibility with HepG2 cells indicates their suitability for non-cancerous applications. Surface modifications in ZnOB and ZnOT, such as BSA and TEOS coatings, reduce toxicity by stabilizing the nanoparticles and limiting ROS generation, which is particularly beneficial for therapeutic safety.

The IC_50_ values were calculated for the different forms of ZnO and reported in Table 3. This table summarizes the IC50 values of ZnO, ZnOT, and ZnOB against two cell lines, HepG2 and Caco-2. The IC50 values indicate the concentration required to inhibit 50% of cell viability, highlighting the selective cytotoxic effects of these nanoparticles on Caco-2 cells while maintaining biocompatibility with HepG2 cells. Additionally, the cytotoxic effects of the ZnO, ZnOB, and ZnOT nanoparticles against the Caco-2 cells were dependent on the nanoparticle concentration and treatment duration. ZnO NPs are known to have selective anticancer activity by inducing a strong electrostatic interaction with the cancer cells^57^. The mechanism of the selective anticancer activity may depend on many variables such as the enzymatic activity of zinc, the functional characteristics of the cells, or particle interactions with the cells. As observed from the obtained data, ZnO had a clear toxic effect on the Caco-2 cells at concentrations an order of magnitude lower than that of HepG2 cells. Our findings are consistent with previous research indicating that the selective cytotoxicity of ZnO nanoparticles against cancer cells can be attributed to the generation of ROS^2^. The overproduction of ROS may also result in oxidative DNA damage and autophagic cell death^58,59^. These mechanisms are particularly relevant in the context of our study, as they explain the observed selective cytotoxicity of ZnO nanoparticles towards Caco-2 cells compared to HepG2 cells. Additionally, the selective cytotoxicity of ZnOB and ZnOT towards cancer cells, particularly at higher concentrations, can be also attributed to mechanisms such as ROS overproduction and oxidative DNA damage. These mechanisms align with existing literature on nanoparticle-based therapies and position ZnOB and ZnOT as superior alternatives to conventional therapies due to their enhanced biocompatibility and targeted action.

**Table 3:** IC50 values of ZnO, ZnOT, and ZnOB nanoparticles in HepG2 and Caco-2 cell lines.

NPs	IC50 (HepG2 Cells)	IC50 (Caco-2 Cells)
ZnO	2080 µg/ml	219 µg/ml
ZnOT	2178 µg/ml	235 µg/ml
ZnOB	1912 µg/ml	308 µg/ml

To provide a clear comparison of ZnO, ZnOB, and ZnOT nanoparticles, Table 4 summarizes their size, zeta potential, and cytotoxicity across different cell lines.

**Table 4:** Summarizing particles size, zeta potential, and cytotoxicity results of ZnO, ZnOB, and ZnOT nanoparticles across different cell lines.

Parameter	ZnO	ZnOB	ZnOT
Size (nm)	140 ± 13.6	342 ± 8.4	145 ± 23.8
Zeta Potential (mV)	-19.21 ± 3.38	-3.24 ± 4.3	19.3 ± 4.16
Cytotoxicity (IC50, µg/mL)			
HepG2	2080	1912	2178
Caco-2	219	308	235

### Live/dead cell assay of ZnO, ZnOB, and ZnOT nanoparticles

**Fig. 6:**
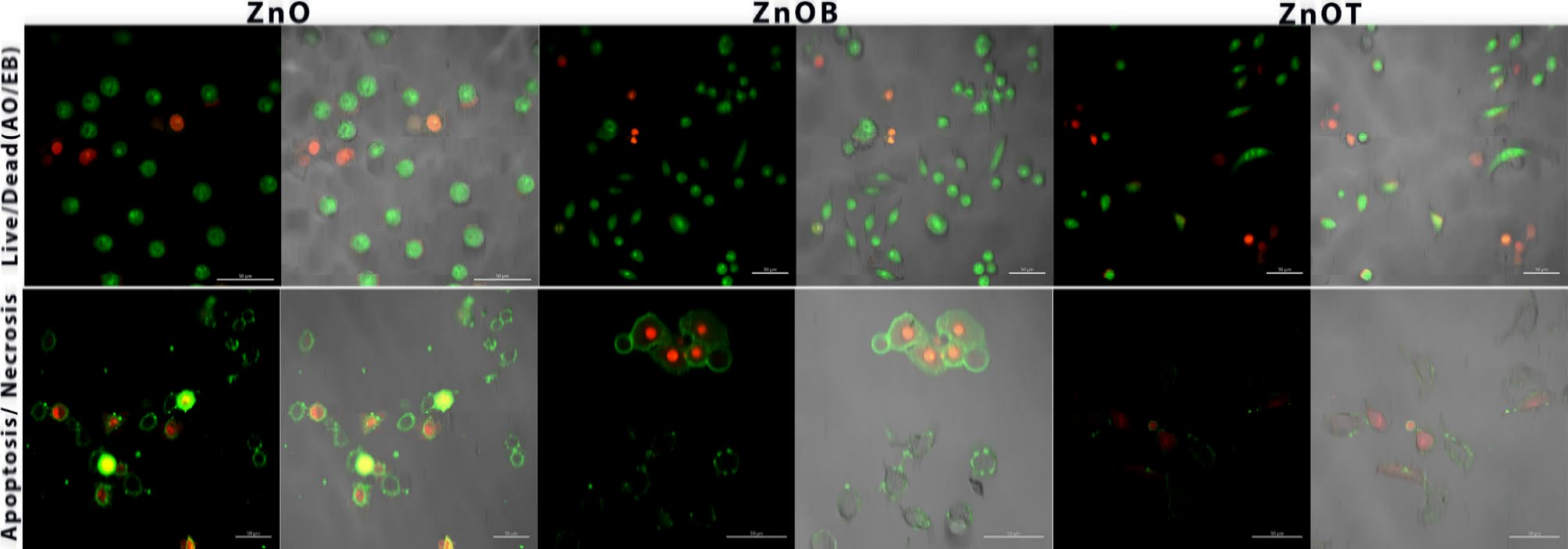
(**a**) CLSM images of live/dead staining using AO and EB on Caco-2 cells after incubating for 24 h with ZnO, ZnOB, and ZnOT nanoparticles, showcasing cell viability and selective cytotoxic effects. (**b**) Annexin V and PI staining images represent apoptosis and necrosis in Caco-2 cells, highlighting the mode of cell death induced by nanoparticles. These findings are crucial for understanding their targeted therapeutic mechanism.

Live dead assays work by employing two dyes. First, AO can penetrate intact cell membranes and intercalate into double-stranded (ds) DNA, emitting green fluorescence and signaling a live cell. Second, EB can only penetrate impaired cell membranes, emitting red fluorescence when bound to DNA signaling a dead cell. The live/dead assay revealed that all the different forms of ZnO induced cell death (Fig. 6a).

### Apoptosis and necrosis assay by CLSM

ZnO nanoparticles induce apoptosis through several mechanisms, including the generation of reactive oxygen species (ROS), disruption of mitochondrial function, and activation of apoptotic pathways. The increase in ROS levels leads to oxidative stress, damaging cellular components and triggering the intrinsic apoptotic pathway. Key markers of apoptosis, such as changes in mitochondrial membrane potential (ΔΨ_m_), are observed in cells exposed to ZnO NPs. The effect of functionalized ZnO NPs on apoptosis can vary with the type of functionalization. Functional groups that enhance ROS generation may intensify apoptotic effects, while those with antioxidant properties may reduce apoptosis. For example, in our assays, Caco-2 cells treated with ZnO, ZnOB, and ZnOT nanoparticles showed a decrease in viable cells, an increase in early apoptotic cells, and a minimal presence of necrotic cells (Fig. 6b)^37^. ZnOT specifically resulted in a higher number of late apoptotic cells. This suggests that functionalizing ZnO with tetraethyl orthosilicate (TEOS) heightened its toxic effects by accelerating apoptosis. Necrosis, characterized by uncontrolled cell death, is often linked to severe cellular damage or stress^60^.

ZnO NPs can induce necrosis through mechanisms such as overwhelming oxidative stress, direct membrane damage, or disruption of cellular homeostasis. Our results indicate that the ZnO-NPs induced DNA damage resulting in cell death for a fraction of the treated cells. Repair mechanisms may no longer act effectively, and sub cytotoxic repairable effects may be converted into cytotoxic effects, leading to a higher percentage of eliminated cells. In addition to NP concentration, the shape, dispersion, and surface charge seemed to play important roles in nanoparticle cytotoxicity^61^.

### Apoptosis necrosis assay by flow cytometry

**Fig. 7:**
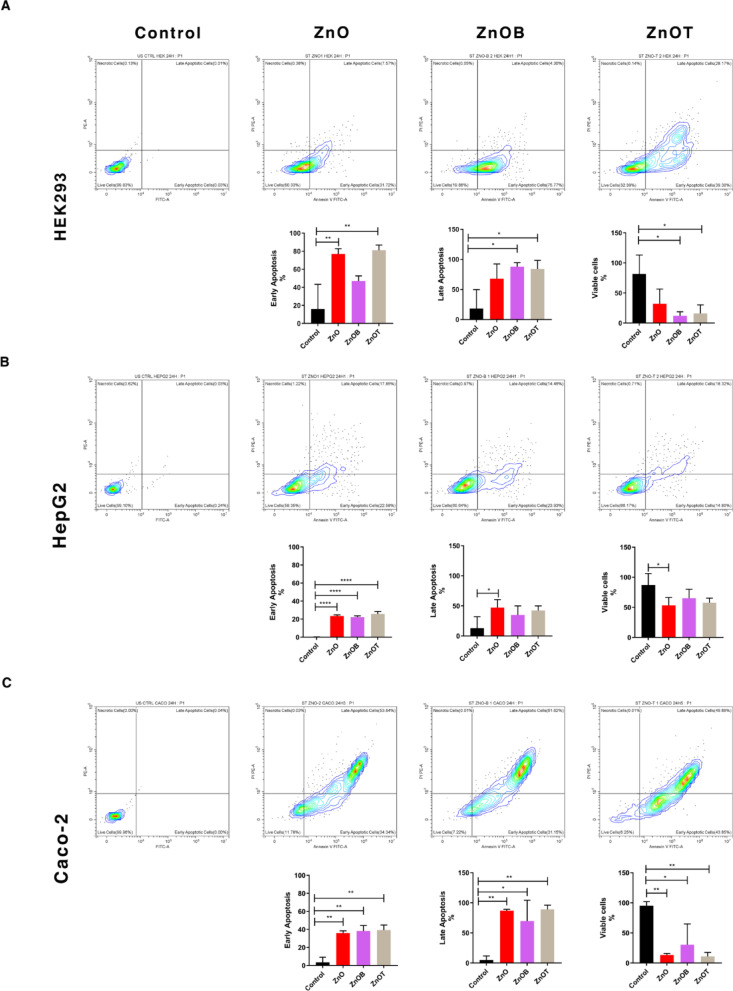
Flow cytometric representative with quantitative statistical analysis of cell apoptosis using Annexin V-FITC/PI staining of three different cell lines: (**a**) HEK293, (**b**) HepG2, and (**c**) Caco-2 under four experimental conditions: untreated control, and treated with ZnO, ZnOB, or ZnOT nanoparticles. This data provides a comparative understanding of apoptosis induction and differential effects across cell lines, reinforcing the nanoparticles’ selective action.

The impact of NPs on cell apoptosis was examined across different cell lines as shown in (Figs. 7). A representative flow cytometric analysis in Fig. 7 showed the gating strategy and the cell populations as the following: viable cells “Annexin V^−^/PI^−^”, early apoptotic cells “Annexin V^+^/PI^−^”, late apoptotic cells “Annexin V^+^/PI^+^” and necrotic cells “Annexin V^−^/PI^+^”.

A statistical analysis of cell apoptosis and viability in the three mentioned cell lines was represented in Fig. 7 with statistical significance indicated by asterisks. The analysis in Fig. 7a showed that in HEK293 cells, the control group exhibited complete cell viability with minimal percentage of apoptosis. ZnO treatment led to a significant increase in early apoptosis and a considerable non-significant increase in late apoptosis. ZnOB treatment showed a slight non-significant decrease in cell viability compared to ZnO and a significant decrease in cell viability compared to the control cells, but still resulted in a non-significant increase in early apoptosis and a significant increase in late apoptosis compared to the control cells. ZnOT treatment caused a significant reduction in cell viability and significant increases in both early and late apoptosis, respectively, indicating its high cytotoxic effect on HEK 293 cells.

In Fig. 7b, the control cells of HepG2 also showed a complete cell viability. The ZnO treated cells showed a significant decrease in cell viability compared to the control cells, while showed significant increases in both early and late apoptosis, respectively. ZnOB treated cells exhibited a non-significant decrease in cell viability compared to the control cells, while showed a non-significant increase in viability compared to ZnO treated cells. On the other hand, ZnOB treated cells showed a significant increase in early apoptosis compared to the control cells. In addition, ZnOT while showing slightly less toxicity than ZnOB, it still showed a non-significant decrease in cell viability compared to the control cells while showed a slight non-significant increase in cell viability compared to ZnO. Moreover, ZnOT caused a significant increase in early apoptosis and a considerable increase in late apoptosis compared to the control cells.

In Fig. 7c, the control group of Caco-2 cells maintained almost complete cell viability. However, ZnO treated cells showed significant decrease in cell viability, and significant increases in early and late apoptosis. ZnOB treatment had a relatively similar impact, it induced a significant decrease in cell viability but slightly less than the ZnO treatment, and it induced a significant increase in early and late cell apoptosis, respectively, compared to the control group. ZnOT also resulted in a significant decrease in cell viability, while showed significant increases in both early and late apoptosis of Caco-2 cells. Finally, ZnOT nanoparticles demonstrated the highest cytotoxicity across all cell lines, strongly inducing apoptosis. ZnOB nanoparticles exhibited moderate cytotoxic effects that varied between cell lines. These findings suggest that the specific composition and surface properties of the nanoparticles significantly influence their cytotoxicity and biological interactions.

### Cell cycle analysis

**Fig. 8:**
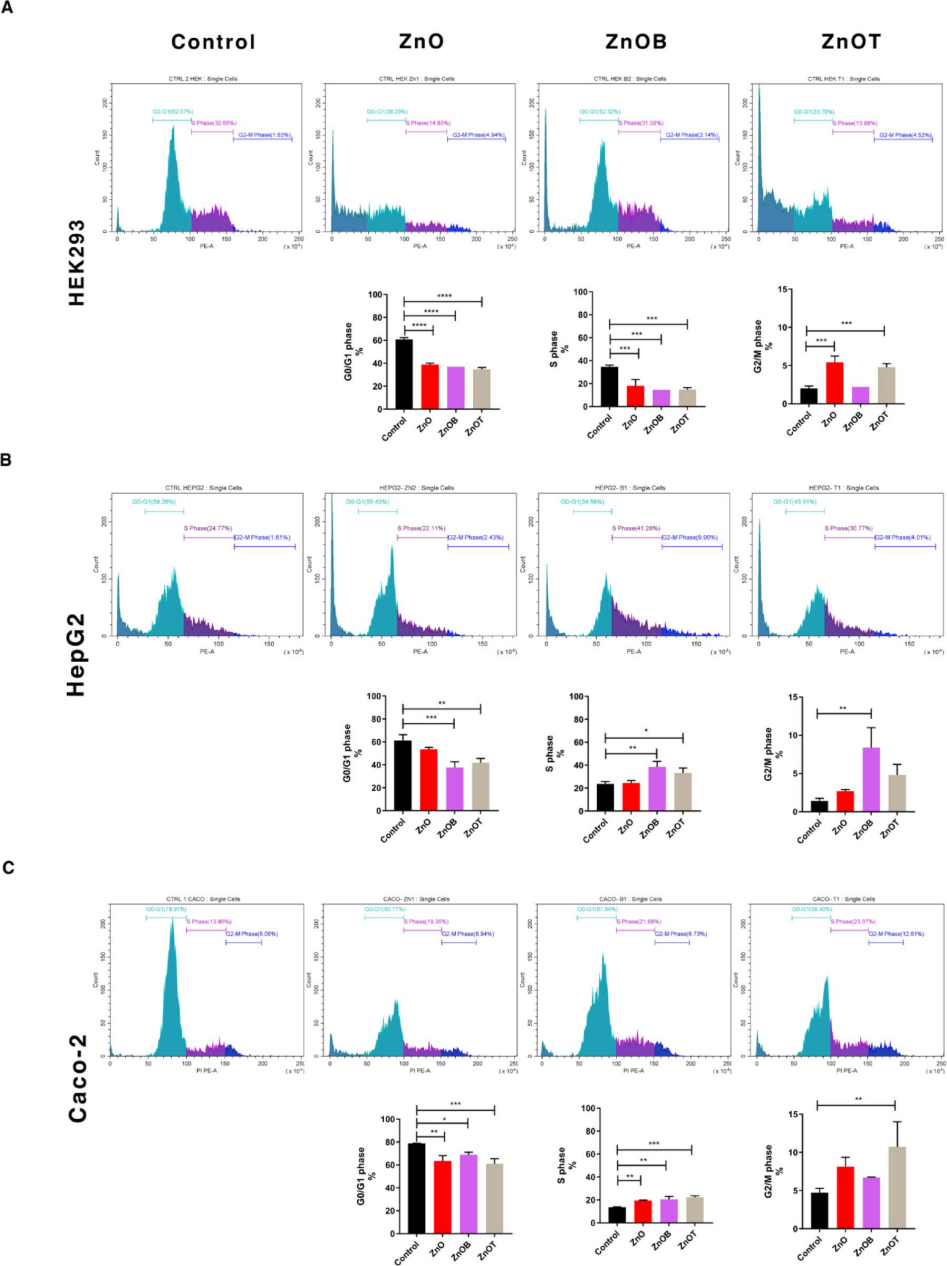
Cell cycle analysis of HEK293, HepG2, and Caco-2 cells after treatment with ZnO, ZnOB, and ZnOT nanoparticles. Representative histograms display cell distribution across G0/G1, S, and G2/M phases for each nanoparticle treatment compared to control. ZnO, ZnOB, and ZnOT induced significant cell cycle disruptions, with ZnOT showing the most pronounced G2/M phase arrest, especially in Caco-2 cells. In HEK293 and HepG2 cells, treatments led to reductions in G0/G1 and S phase populations, indicating cell cycle progression interference across all tested cell lines.

The impact of NPs on cell cycle was examined across the different cell lines. A representative histogram in Fig. 8 showed the cell cycle stages (G0/G1-S-G2/M) of each experimental condition and showed the progression of the cell cycle across the Caco-2 cell lines upon the effect of the tested NPs. The statistical analysis of cell cycle in the three cell lines, (HEK293, HepG2, and Caco-2), were represented in Fig. 8. Figure 8a, for HEK293 Cells, the control untreated cells showed a predominant increase of the cells in the G0/G1 phase, and showed fewer cell number in the S phase, and the fewest cell number in G2/M phase. Treatment with ZnO, ZnOB, and ZnOT significantly decreased the number of cells in the G0/G1 phase as compared to the control cells. ZnO and ZnOB and ZnOT treatments caused significant decreases in the number of the cells in the S phase as compared to the control cells. ZnO and ZnOT both resulted in significant increases in the G2/M phase as compared to the control group, suggesting a potential block in the transition from G2 to mitosis. As shown in Fig. 8b, for HepG2 Cells, ZnO caused a non-significant decrease in the number of the cells in G0/G1 phase as compared to the control cells. ZnOB and ZnOT treatments significantly reduced the number of the cells in G0/G1 phase, respectively. In addition, ZnO treatment led to a slight non-significant increase in the S phase, while ZnOB and ZnOT significantly increased the number of the cells in S phase, respectively, as compared to the control cells. As compared to the control cells, ZnO showed a slight non-significant increase in the number of the cells in G2/M phase, while ZnOB significantly increased the number of the cells in G2/M phase, and ZnOT treatment caused a non-significant increase in the number of the cells in G2/M phase.

As shown in Fig. 8c, for Caco-2 cells, ZnO, ZnOB, and ZnOT treatments significantly reduced the number of the cells in the G0/G1 phase, respectively, compared to the control cells. The number of the cells in S phase significantly decreased upon ZnO, ZnOB, and ZnOT treatments, respectively, compared to the control cells. ZnOT treatment only showed a significant increase in the G2/M phase compared to the control cells while ZnO showed a slight increase in the G2/M phase compared to the control cells. All prior results indicate that NPs cause notable changes in cell cycle progression across all tested cell lines, consistently showing a reduction in the G0/G1 phase alongside increases in the S and G2/M phases. Notably, ZnOT exhibits the most significant impact, implying it may greatly disrupt normal cell cycle progression, potentially resulting in growth inhibition and cytotoxic effects.

### Cellular uptake and internalization of the zinc oxide nanoparticles by CLSM

**Fig. 9:**
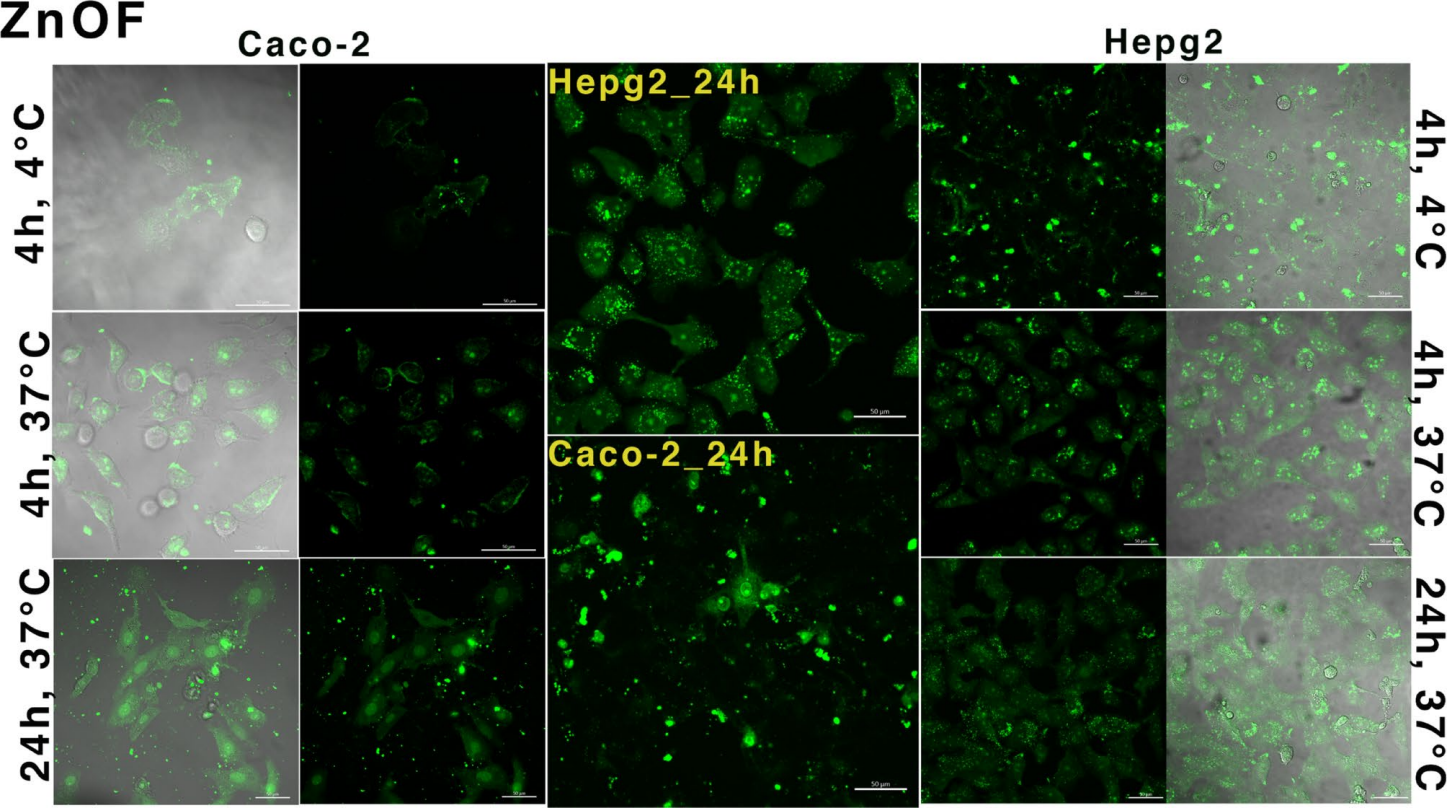
CLSM imaging of ZnOF nanoparticle internalization in Caco-2 and HepG2 cells. Fluorescence images demonstrating temperature-dependent internalization patterns in Caco-2 cells at different time points (**a**–**c**) and 24 h uptake in (A) HepG2 and (B) Caco-2 cells, with localization into cytoplasm and nuclei.

ZnO NPs resulted in measurable uptake being detected in almost all cells after 4 h of incubation (Fig. 9). Furthermore, individual cell images revealed that the ZnO NPs were internalized into the cell cytoplasm and nucleus (Fig. 9)^42^. Due to the negative zeta potential, ZnO could form new pores or channels in the cell membrane and nucleus, enabling it to be internalized into cells. Figure 13 shows the localization of ZnO in the cytoplasm and nucleus of the HepG2 and Caco-2 cells. Clearly, ZnO could be taken up by the nucleus and endosomes as its uptake by mitochondria may be one of the reasons that it has a rapid effect on high metabolism cancer cells due to it affecting mitochondrial functionality. Additionally, uptake through endosomes was assumed due to the presence of small vesicles surrounding the nucleus after incubation for 4 h at 37 °C, which did not appear after incubation for 24 h at 37 °C. After the 24 h, the nucleus of the Caco-2 cells had been denatured, and the cells had begun to undergo cell death^62,63^. Caco-2 cells, resembling enterocytes, focus on nutrient absorption, digestion, and active transport. Their oxidative stress enzymes help detoxify foreign substances, but when exposed to ZnO nanoparticles, they often exhibit uptake, stress, and inflammation due to their specialized pathways for xenobiotic absorption^64,65^. HepG2 cells, with robust xenobiotic-metabolizing enzymes like cytochrome P_450_, excel at processing foreign substances. Their role in liver detoxification makes them efficient in metabolizing ZnO nanoparticles, leading to oxidative stress and cytotoxicity as they break down and biotransform these particles^66,67^.

**Fig. 10:**
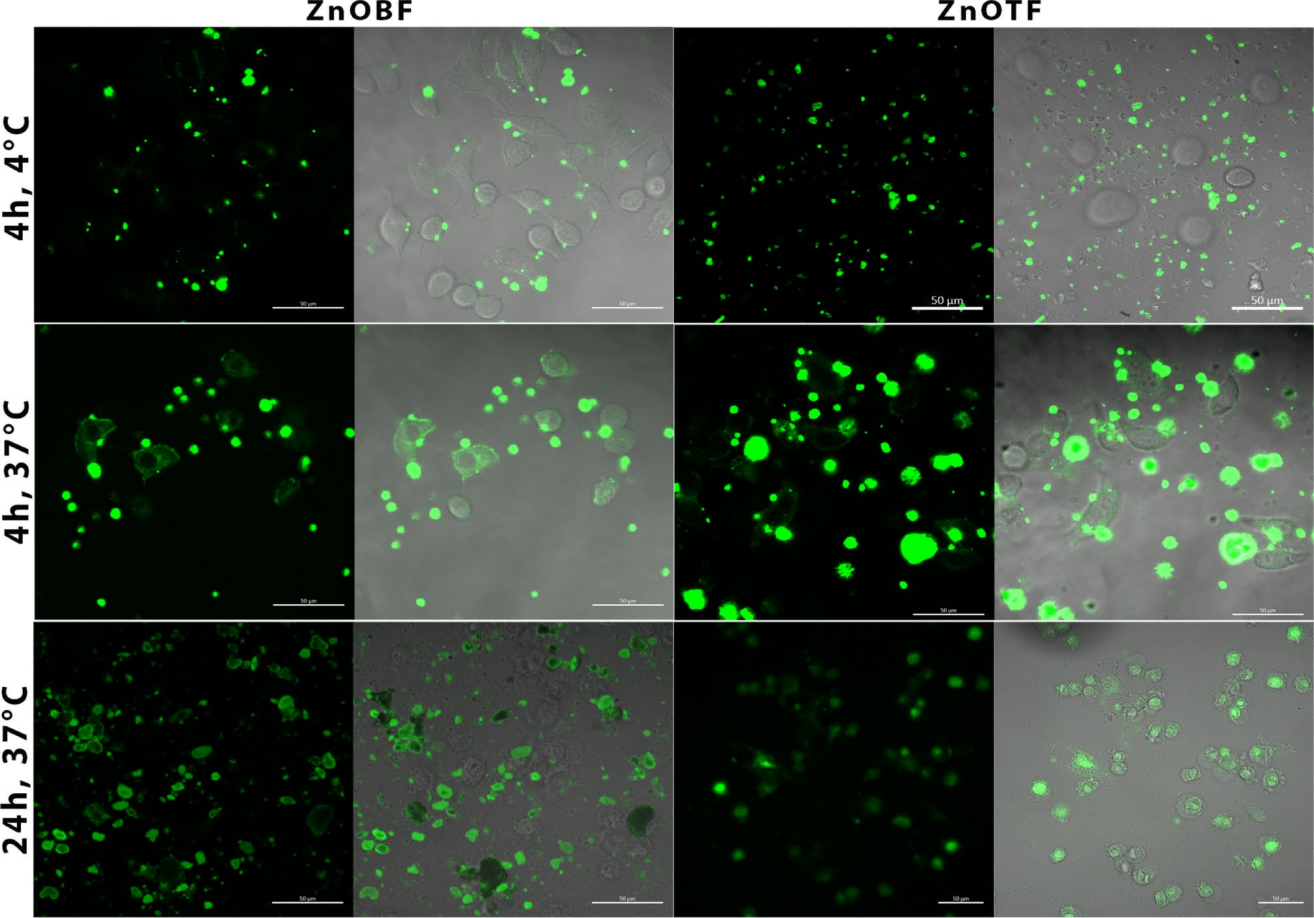
CLSM imaging of ZnOBF and ZnOTF uptake in Caco-2 cells. Fluorescence images showing uptake at (**a**) 4 °C for 4 h, (**b**) 37 °C for 4 h, and (**c**) 37 °C for 24 h. FITC-labeled nanoparticles exhibit enhanced cytoplasmic internalization at higher temperatures and longer exposure durations.

On the other side, the ZnOB and ZnOT nanoparticles showed poor cellular uptake, internalization into the cytoplasm, and attachment to the Caco-2cell membrane after incubating for 4 h at 4 °C (Fig. 10). However, their uptake was enhanced by incubating for either 4 h at 37 °C or 24 h at 37 °C (Fig. 10). Finally, cellular uptake and internalization of the prepared nanoparticles indicated that ZnO had entered the cytoplasm and nucleus after 4–24 h at 37 °C (likely interacting with DNA strands), while the modified ZnOB and ZnOT nanoparticles had only diffused into the cytoplasm and did not enter the nucleus. These results suggest that although TEOS and BSA enhanced the dispersibility and stability of ZnO in solution. Zn entered cells in the ionized form, Zn^2+^, thus maintaining its same behavioral effect on cancer cells after modification, as shown in the cytotoxicity assay test. This finding likely reflected the fact that the nuclear pores are typically 20–50 nm in size, limiting the access of the ZnOB and ZnOT nanoparticles, which have a diameter greater than that of ZnO^68^. This indicated that the different forms of ZnO are enter the cells through diffusion and their uptake may be increased through other transport processes such as endocytosis or phagocytotic transport.

### Cellular uptake and internalization of the zinc oxide nanoparticles flow cytometry

**Fig. 11:**
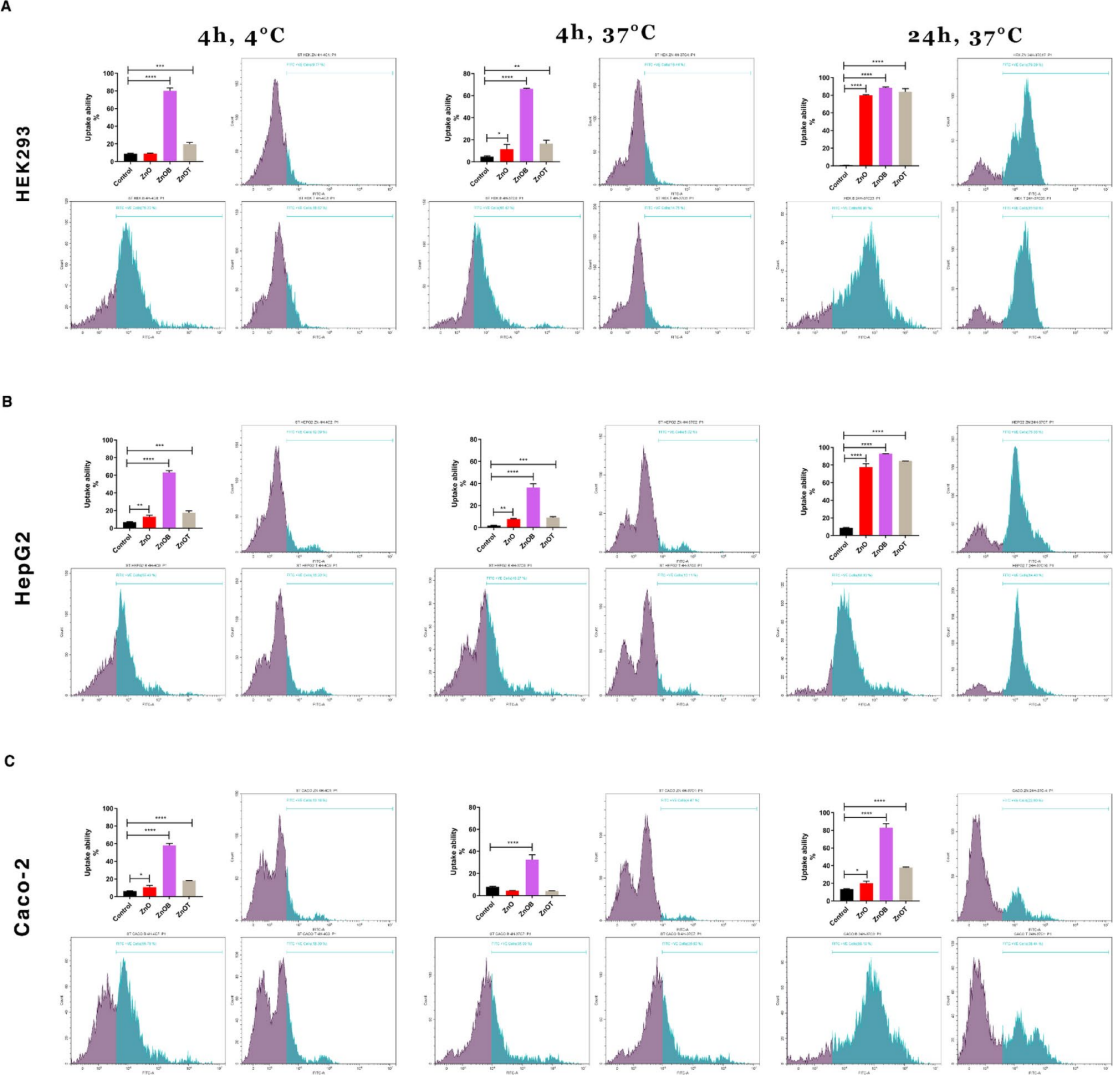
Flow cytometry-based quantification of nanoparticle uptake in HEK293, HepG2, and Caco-2 cells. Uptake efficiency of ZnO, ZnOB, and ZnOT nanoparticles in (**a**) HEK293, (**b**) HepG2, and (**c**) Caco-2 cells under different temperature and incubation conditions, demonstrating time- and temperature-dependent internalization patterns.

For HEK293 Cells (Fig. 11a), when the cells incubated at 4 °C for 4 h, ZnOB treated cells nanoparticles exhibited the highest uptake, as compared to the control cells, followed by ZnOT which also exhibited also a high uptake compared to the control cells while ZnO treated cells showed non-significant uptake, compared to the control cells. This suggests that ZnOB has a strong affinity for HEK293 cells, potentially involving an energy-independent uptake mechanism. When the cells incubated at 37 °C for 4 h, ZnOB also showed the highest uptake, followed by ZnO and ZnOT that both showed significant increased uptakes compared to the control cells, indicating that ZnOT uptake may be temperature-dependent and likely involves an active process. After 24 h at 37 °C, ZnO, ZnOB and ZnOT achieved significantly increased uptake compared to the control cells, suggesting that HEK293 cells reach maximum uptake capacity over this extended period. For HepG2 Cells (Fig. 11b), when the cells were incubated at 4 °C for 4 h, ZnOB had the most significant increased uptake followed by ZnOT, and ZnO as compared to the control cells. The overall uptake was lower compared to HEK293 cells, indicating different uptake mechanisms or less affinity in HepG2 cells. when the cells were incubated at 37 °C for 4 h, ZnOB still had the most significant increased uptake, followed by ZnO and ZnOT which still showed significant increased uptakes. After 24 h at 37 °C, similar to HEK293 cells, ZnO, ZnOB, and ZnOT achieved significantly increased uptake compared to the control cells. indicating that HepG2 cells also achieve saturation with sufficient time.

For Caco-2 Cells (Fig. 11c), when the cells were incubated at 4 °C for 4 h ZnOB showed the most significant increased uptake followed by ZnOT and ZnO, respectively. At 37 °C for 4 h, ZnOB still had the most significant increased uptake while ZnO and ZnOT both showed non-significant uptake compared to the control cells. After 24 h at 37 °C, ZnOB showed the most significant increased uptake, followed by ZnO and ZnOT which both showed significantly increased uptakes, respectively as compared to the control cells, indicating saturation with sufficient incubation time. Finally, ZnOB nanoparticles demonstrated the highest uptake across all cell lines and conditions, indicating greater cellular affinity or more efficient internalization, potentially due to enhanced surface modifications. ZnO nanoparticles showed lower uptake, especially at shorter time points, suggesting they may need more time or rely on energy-dependent mechanisms. ZnOT nanoparticles displayed intermediate uptake levels, with increased uptake observed with higher temperatures and longer incubation times, indicating a role of active, temperature-dependent processes. These findings provide valuable insights into the differential uptake abilities of the nanoparticles, guiding their potential applications based on cell targeting and uptake efficiency.

### Mitochondrial function assay

**Fig. 12:**
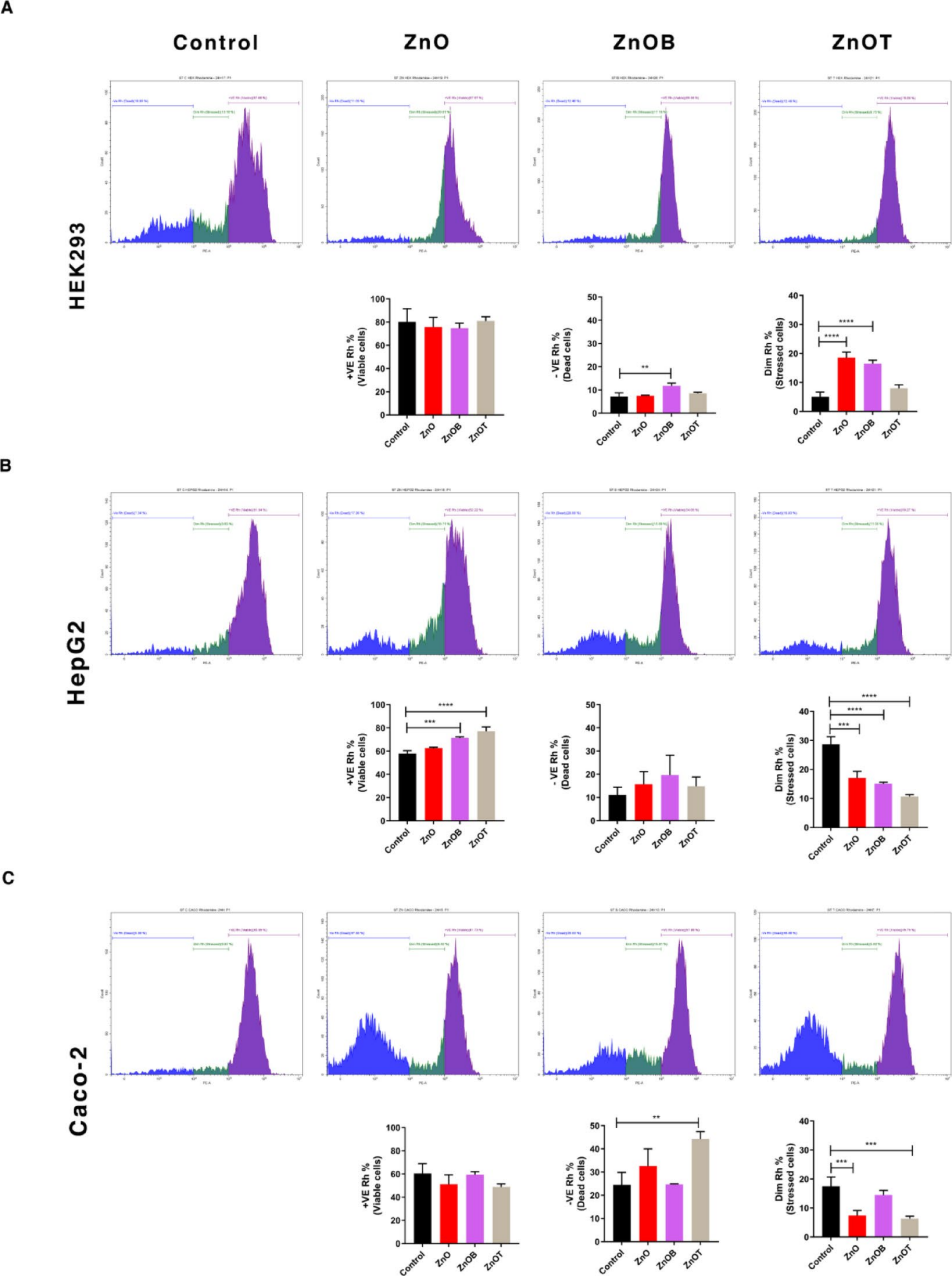
Mitochondrial membrane potential assessment using Rhodamine 123 staining. Flow cytometric analysis of live, dead, and stressed cells in HEK293, HepG2, and Caco-2 lines after nanoparticle exposure, linking mitochondrial function disruption to nanoparticle-induced cytotoxicity.

The observed differences in cell viability, stress, and cytotoxicity among the nanoparticles can be understood through their effects on mitochondrial membrane potential (MMP) (Fig. 12). For HEK293 Cells, the significant increases in dead cells upon ZnOB treatment, and stressed cells upon ZnO and ZnOB treatments, together with the decrease in the percent of dead and stressed cells upon ZnOT treatment, suggests that these nanoparticles may influence mitochondrial function. ZnO, with its higher cytotoxicity, could be causing a substantial loss in MMP, leading to mitochondrial dysfunction, increased oxidative stress, and ultimately cell death. ZnOB’s increase in stressed cells, but not as severe as ZnO, may also reflect moderate mitochondrial impairment. In contrast, ZnOT’s relatively better biocompatibility might be associated with less disruption of MMP, thus contributing to reduced cell stress and apoptosis. ZnOB and ZnOT showed higher cell viability and lower levels of stress compared to ZnO for HepG2 Cells. This differential response could be related to how each nanoparticle affects MMP. ZnOB and ZnOT likely maintain better MMP compared to ZnO, thereby supporting mitochondrial health and reducing stress-induced apoptosis. ZnO, which induces higher stress and lower viability, might compromise MMP significantly, leading to mitochondrial damage and cell death. The observed protection offered by ZnOB and ZnOT may be linked to their ability to preserve MMP and mitochondrial function. The reduction in stressed cells observed with NPs, coupled with minimal cytotoxic effects, indicates that these nanoparticles might better preserve MMP in Caco-2 cells. This preservation of MMP could contribute to reduced oxidative stress and enhanced cell viability. ZnO’s moderate stress response and cytotoxicity could be associated with partial disruption of MMP, leading to increased cell stress and death. Our results confirm that the differential effects of ZnO, ZnOB, and ZnOT on cell viability, stress, and cytotoxicity are likely related to their impact on mitochondrial membrane potential. ZnO, with higher cytotoxicity and stress, may cause significant disruption of MMP, leading to mitochondrial dysfunction and cell death. ZnOB, exhibiting better biocompatibility, might maintain a more stable MMP, thus supporting cell viability and reducing stress. These findings emphasize the importance of mitochondrial health in mediating the cellular responses to different nanoparticles and highlight the potential for ZnOB to offer better safety profiles due to their lesser impact on mitochondrial function.

Despite the promising anticancer activity of ZnO nanoparticles demonstrated in this study, their clinical translation faces notable challenges rooted in aggregation tendencies and biocompatibility concerns. A limitation of unmodified ZnO NPs is their propensity to aggregate in biological media, driven by high surface energy and interactions with proteins or ions, which compromises colloidal stability, biodistribution, and therapeutic efficacy^22,29^. Aggregation reduces the effective surface area available for drug delivery or ROS generation and may lead to unintended accumulation in non-target tissues, as observed in prior studies^20^. Furthermore, while ZnO NPs exhibit pH-selective cytotoxicity, residual Zn^2+^ release in physiological conditions (pH 7.4) can induce off-target toxicity in normal cells, as evidenced by moderate HEK293 cell viability reduction (IC ~ 50 ~ = 2080 µg/mL)^30^. This underscores the need for precise surface engineering to enhance biocompatibility without compromising anticancer activity. In this study, BSA and TEOS coatings addressed these limitations by sterically stabilizing nanoparticles (PDI < 0.4) and reducing Zn^2+^ leakage, as confirmed by zeta potential and cytotoxicity assays. However, long-term stability under physiological conditions, immune response modulation, and scalable synthesis of uniform coatings remain unresolved challenges^9^. Future work must also evaluate in vivo clearance pathways and chronic toxicity to fully realize the clinical potential of ZnO-based nanotherapies.

## Conclusion

This study thoroughly characterizes zinc oxide nanoparticles (ZnO NPs) and their modified forms, ZnOB and ZnOT, which are coated with bovine serum albumin (BSA) and tetraethyl orthosilicate (TEOS), respectively. These coatings enhance the stability, dispersibility, and functionality of ZnO NPs. UV-Vis absorption spectra showed slight shifts with coatings, confirming minimal impact on particle size, while FITC labeling and FTIR verified successful functionalization. Cytotoxicity assays revealed ZnO’s selective toxicity toward Caco-2 cancer cells at lower concentrations, highlighting its potential as a targeted cancer therapy. ZnOB and ZnOT demonstrated improved stability, dispersibility, and controlled release capabilities, making them suitable for enhanced cellular interactions. Apoptosis assays indicated ZnOT induced the highest levels of early and late apoptosis, with cell cycle analysis showing significant disruption by ZnOT. Mitochondrial function assays confirmed ZnOT’s strong impact on mitochondrial membrane potential, correlating with increased cytotoxicity. Cellular uptake studies showed ZnOB had the highest uptake, with both ZnOB and ZnOT demonstrating better biocompatibility and stability than unmodified ZnO, making them suitable for drug delivery, imaging, and antimicrobial applications. In summary, ZnO NPs are promising for targeted cancer therapy, while ZnOB and ZnOT offer enhanced stability, uptake, and biocompatibility, supporting their potential in diverse biomedical applications. Future work will evaluate their long-term stability and explore further functionalization strategies.

## Data Availability

The data used during the current study are available from the corresponding author upon reasonable request.
